# Late-Season Sweet Orange Selections Under Huanglongbing and Citrus Canker Endemic Conditions in the Brazilian Humid Subtropical Region

**DOI:** 10.3389/fpls.2022.915889

**Published:** 2022-05-31

**Authors:** Deived Uilian de Carvalho, Carmen Silvia Vieira Janeiro Neves, Maria Aparecida da Cruz, Talita Vigo Longhi, Franklin Behlau, Sérgio Alves de Carvalho, Rui Pereira Leite Junior

**Affiliations:** ^1^Área de Proteção de Plantas, Instituto de Desenvolvimento Rural do Paraná (IDR-Paraná), Londrina, Brazil; ^2^Centro de Ciências Agrárias, Universidade Estadual de Londrina (UEL), Londrina, Brazil; ^3^Departamento de Pesquisa e Desenvolvimento, Fundo de Defesa da Citricultura (Fundecitrus), Araraquara, Brazil; ^4^Centro de Citricultura “Sylvio Moreira” Instituto Agronômico de Campinas (IAC), Cordeirópolis, Brazil

**Keywords:** *Citrus × sinensis* (L.) Osbeck, late-season maturing, fruit quality and yield, tree size, disease incidence, orchard diversification

## Abstract

The Brazilian citrus orchards are comprised by few genotypes, which increases the risk of pest and disease outbreaks. The diversification of sweet oranges (*Citrus × sinensis*) in orchards also generates off-season revenue and extend the fruit processing period. This study aimed to evaluate several horticultural traits of 19 late-season sweet orange selections under citrus canker and huanglongbing (HLB) endemic condition in northwestern Paraná state, Brazil, in a long-term field experiment. Tree size, yield, fruit quality for fresh fruit and industrial markets, estimates of tree density and yield, and citrus canker and huanglongbing (HLB) incidences were assessed. The experimental design was a randomized block with three replicates and five trees per unit. The orchard was drip-irrigated and arranged at tree spacing of 6.5 m × 4.5 m. All scions were graft-compatible with Rangpur lime (*C. × limonia*). Valencia selections had the tallest trees and largest canopies, particularly Olinda, Frost and #121 with heights and volumes greater than 4.20 m and 43 m^3^, respectively. Natal África do Sul and Whit’s Late Valencia trees were the most productive with cumulative yields above 640 kg per tree. Most of the selections produced fruits of excellent physicochemical quality attending the fresh fruit and industrial market requirements. All selections showed similar horticultural characteristics for the fresh market, while Natal África do Sul and Charmute de Brotas were more suitable for juice processing. Frost Valencia and Valencia Late Fla. had the highest incidence of citrus canker on fruits (>20%), whereas IPR Folha Murcha, Charmute de Brotas and some Valencia selections (Chafeei Late, Campbell 479, Campbell 294, Olinda, Mutação and Whit’s Late) exihibed low incidence (3.0–17.7%). At 9 years, Valencia Mutação trees had high HLB incidence (93%). In contrast, Natal IAC and Folha Murcha IAC showed the lowest HLB incidence (13%). Our results revealed that Natal IAC, Folha Murcha IAC, IPR Folha Murcha, Natal Murcha, Campbell 479 Valencia and Valencia Late Fla. had the best horticultural performance in addition to low HLB incidence. Together, these late-season sweet oranges are the most advantageous selections for citrus orchard diversification under citrus canker and HLB endemic conditions in humid subtropical regions.

## Introduction

Brazil is the world’s largest producer of sweet oranges [*Citrus × sinensis* (L.) Osbeck], accounting for around one-quarter of the global fruit production and three-quarters of the global orange juice exports (FAO, 2019; Spreen et al., 2020; U.S. Department of Agriculture, 2021). In 2020, the Brazilian production of sweet oranges was 17 M tons, followed by India with 9.9 M tons, China with 7.5 M tons and United States with 4.8 M tons (FAO, 2019). Citrus producing areas in Brazil is concentrated in the state of São Paulo, where sweet oranges are mainly produced for juice processing (~70%), followed by Minas Gerais, Bahia, and Paraná (IBGE, 2020; Spreen et al., 2020; U.S. Department of Agriculture, 2021).

In the last decades, advanced cultural practices in the citrus industry have been of utmost importance for Brazil to maintain the leadership in the global sweet orange production (FAO, 2019; Carvalho et al., 2019a; Bassanezi et al., 2020). These practices include irrigation, fertilization, high tree density, pest and disease controls, and planting of citrus cultivars that are more productive and adapted to a wide range of environmental conditions (Carvalho et al., 2019a; Bassanezi et al., 2020; Behlau et al., 2021; Girardi et al., 2021). The presence and progression of citrus diseases in orchards are frequent under the humid subtropical climate (Bassanezi et al., 2020; Carvalho et al., 2021a). Nevertheless, even under intense disease pressure, such as citrus tristeza virus (CTV), citrus canker, citrus variegated chlorosis (CVC), citrus black spot, leprosis and huanglongbing (HLB, a.k.a. citrus greening), the Brazilian citrus industry has adapted to a challenging scenario relying on an efficient management system (Bassanezi et al., 2020; Spreen et al., 2020).

Citrus canker and HLB are particularly important for the citrus industry in the state of Paraná (Leite Júnior and Mohan, 1990; Sauer et al., 2015). Historically, most of the area of Paraná was prohibited to grow citrus until the late 1980s due to the occurrence of citrus canker (*Xanthomonas citri* subsp. *citri*, *Xcc*) and the lack of efficient control measures by then (Behlau, 2021). In 1990, an integrated disease management program was developed to prevent and control the citrus canker in new areas across the state (Leite Júnior and Mohan, 1990). This program involves some cultural and preventive measures such as planting resistant or less susceptible citrus cultivars to *Xcc*, disease-free nursery trees, establishment of windbreaks, periodic sprays of copper-based bactericides, and control of the citrus leafminer *Phyllocnistis citrella* Stainton (Leite Júnior and Mohan, 1990; Behlau et al., 2021). Canker may cause symptoms in different organs of citrus plants, but lesions on fruits may increase premature fruit drop and reduce the marketability of fresh fruit (Lanza et al., 2019; Behlau, 2021). Citrus cultivars have a broad range of susceptibility to citrus canker, ranging from resistant, as the case of Folha Murcha, to highly susceptible as Hamlin (Amaral et al., 2010; Vargas et al., 2013; Carvalho et al., 2015). Based on previous investigations, only genotypes that show certain resistance to citrus canker are authorized for planting in Paraná, which includes the early-season sweet oranges IAPAR 73, Navelina, Salustiana, and Cadenera; the mid-season Pera, Shamouti and Jaffa; and the late-season Valencia, Folha Murcha and Natal (IAPAR, 1992; Auler et al., 2014). Therefore, planting canker-resistant genotypes has been the most efficient and inexpensive measure to control this disease (Behlau et al., 2021).

More recently, HLB has become the most serious threat to the citrus industry worldwide (Bassanezi et al., 2020). The ‘*Candidatus* Liberibacter asiaticus’ (*C*Las), a phloem-limited bacterium associated with the HLB pathosystem, was first identified in Paraná in 2007 (Nunes et al., 2007; Sauer et al., 2015) and rapidly spread across the main citrus-growing areas of the state (Gottwald, 2010; Paula et al., 2019). Integrated management approaches for mitigating HLB have been established in Brazil such as planting of healthy nursery trees; monitoring of the citrus-growing and surroundings areas; area-wide control of the *C*Las vector Asian citrus psyllid (ACP; *Diaphorina citri* Kuwayama); removal of symptomatic trees; and release of ACP’s parasitoids, as the *Tamarixia radiata* Waterston (Nunes et al., 2010; Bassanezi et al., 2020).

The Brazilian orange belt is composed of a few genotypes, with Hamlin (early-season), Pera (mid-season), and Valencia and Natal (late-season) as the predominant cultivars (Carvalho et al., 2019a). Further, they are highly demanded for juice processing (Carvalho et al., 2019a; Spreen et al., 2020). The late-season Valencia and Natal accounts for at least 55% of the planted sweet orange trees in the Brazilian orchards, as these cultivars are very productive and bear fruit of high juice quality (Spreen et al., 2020). However, the limited number of cultivars in citrus orchards may favor outbreaks of pests and diseases due to the narrow genetic pool (Carvalho et al., 2019a,b, 2021a). Therefore, the diversification of scion and rootstock citrus genotypes is of paramount importance to improve citrus protection. Moreover, this strategy may allow growers to obtain higher profits when the fruit supply is low and increase the options of sweet oranges for the fresh market, besides enabling the industry to extend the juice processing period.

Several citrus scion and rootstock accessions have been introduced and selected in different breeding programs in Brazil. For instance, the Instituto de Desenvolvimento Rural do Paraná (IDR - Paraná) maintains a program (Carvalho et al., 2020) that constantly evaluates potential citrus selections to be included in the state of Paraná, aiming at orchard diversification and production of high-quality fruits for the fresh market and processing (Paula et al., 2022). Within this context, this study reports the evaluation of several horticultural traits of 19 late-season sweet orange selections based on a long-term field experiment established in northwestern Paraná, Brazil. It is also hoped to gain a better understanding of the suitability of selections for the fresh fruit market and/or juice processing, which will contribute to a more strategic and targeted establishment of new orchards.

## Materials and Methods

### Field Location

**Figure 1:**
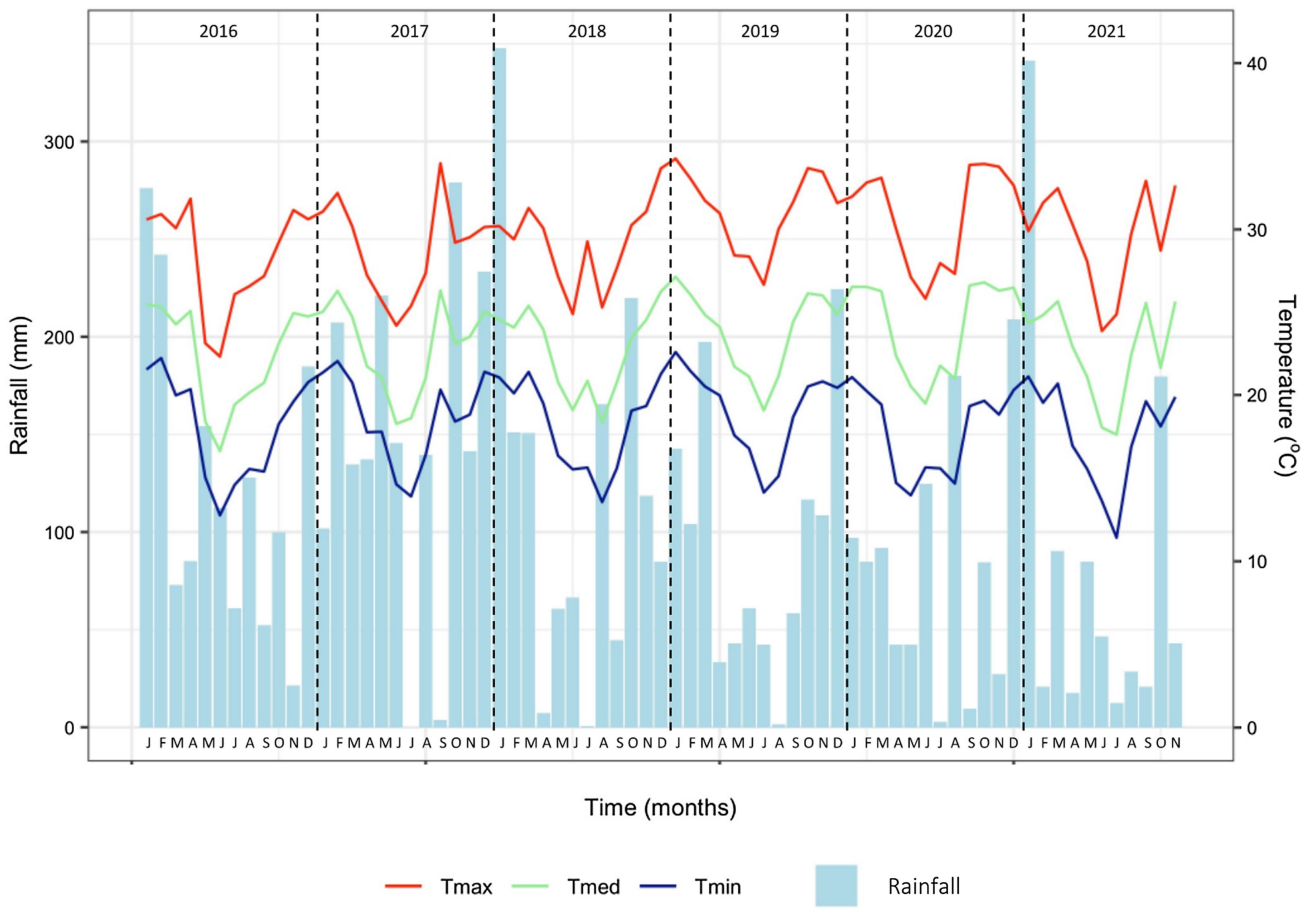
Monthly rainfall and maximum (Tmax), medium (Tmed) and minimum (Tmin) average temperatures from 2016 to 2021 in Guairaçá, state of Paraná, Brazil (Source: IDR–Paraná, 2021).

The experimental orchard was conducted from 2012 to 2021 in the Experimental Station of Cocamar Cooperativa Agroindustrial in the municipality of Guairaçá, state of Paraná, southern Brazil (latitude 22° 56′ 04″ S, longitude 52° 41′ 08″ W, and altitude of 518 m). The climate of the region is humid subtropical (Cfa) according to the Köppen-Geiger climate classification, with annual maximum and minimum temperatures of 28.4 and 17.8°C, respectively, annual average rainfall of 1,527 mm (Figure 1) and relative humidity of 69% (IDR–Paraná, 2021). The soil is a Typic Haplustox with 90% sand, 1% clay, base saturation of 20%, and pH of 3.9 in the 0–40 cm layer (Larach et al., 1984).

### Plant Material

**Figure 2:**
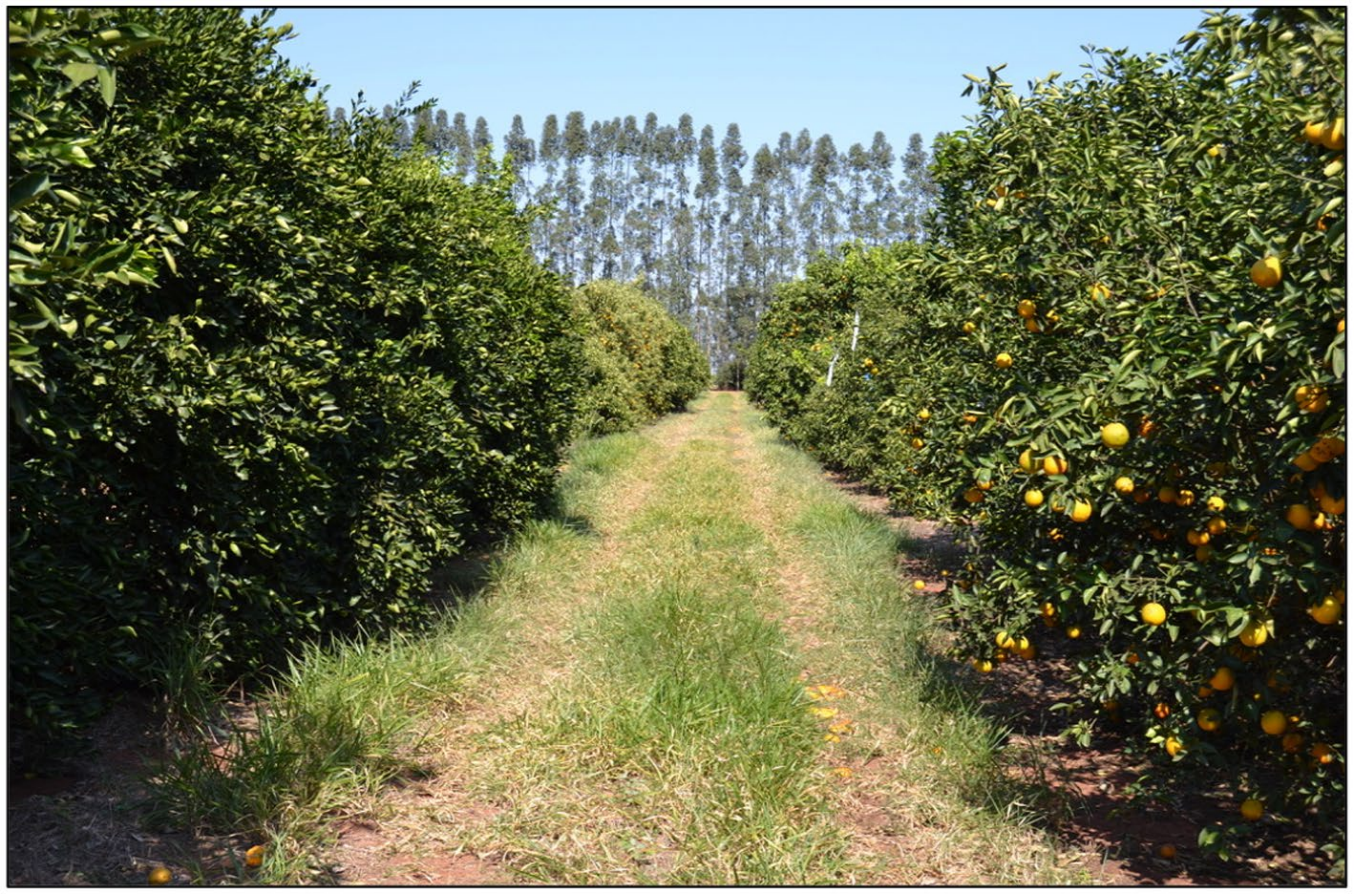
Orchard arrangement of nine-year-old late-season sweet orange selections grafted onto Rangpur lime rootstock with two-row windbreaks of *Eucalyptus grandis* (Hill) ex Maiden (in the back) in Guairaçá, state of Paraná, Brazil.

The nursery trees of the 19 late-season selections of Valencia, Charmute de Brotas, Natal and Folha Murcha sweet orange cultivars were provided by the Citrus Active Germoplasm Bank of the Instituto Agronômico de Campinas – IAC/Centro de Citricultura “Sylvio Moreira” in Cordeirópolis, state of São Paulo, Brazil, and the Instituto de Desenvolvimento Rural do Paraná – IAPAR/EMATER (IDR - Paraná) in Londrina, state of Paraná. The origin of the 19 selections of sweet orange (*C. × sinensis*) evaluated in this study is shown in Table 1. All trees were grafted onto the Rangpur lime [*C. × limonia* (L.) Osbeck] rootstock, which is graft-compatible with scions and adapted to a wide range of soil and climate conditions. The experimental design was a randomized block with 19 scions, three replicates and five trees per plot. The experimental orchard was planted in December 2012 at tree spacing of 6.5 m × 4.5 m corresponding to 341 trees.ha^−1^ (Figure 2). The trial also had sides planted with two-row windbreaks of *Eucalyptus grandis* (Hill) ex Maiden. The arrangement of the windbreak rows was in a zig-zag order at spacing of 2 m between trees and 1 m within rows, and 8 m apart of the experimental orchard.

**Table 1:** Origin of the 19 selections of sweet orange (*Citrus × sinensis*) assessed in this study.

Sweet orange selection	Origin of selection	Material source	Name in the source institution
Whit’s Late Valencia	United States	CCSM^1^	Valencia Whit’s Late IAC 1373
Cutter Valencia	United States	CCSM	Valencia Cutter IAC 1726
Berry Valencia	Australia	CCSM	Berry Valencia IAC 1335
Frost Valencia	United States	CCSM	Valencia Frost IAC 1727
Valencia Mutação	Brazil	CCSM	Valencia IAC 1754
Valencia IAC	Brazil	CCSM	Valencia IAC
Olinda Valencia	United States	CCSM	Valencia Olinda IAC 478
Chafeei Late Valencia	Australia	CCSM	Valencia Chafeei Late IAC 1357
Campbell 479 Valencia	United States	CCSM	Valencia Campbell IAC 1724
Campbell 294 Valencia	United States	CCSM	Valencia Campbell EECB – 294
Valencia Late 161	United States	CCSM	Valencia Late IAC 161
Valencia Late Fla.	United States	CCSM	Valencia Late IAC 1361
Valencia #121	Cuba	CCSM	Valencia IAC 1430
Charmute de Brotas	Brazil	CCSM	Charmute de Brotas IAC 2007
Natal África do Sul	South Africa	CCSM	Natal África do Sul IAC 481
Natal IAC	Brazil	CCSM	Natal IAC
Natal Murcha	Brazil	CCSM	Natal Folha Murcha IAC 491
Folha Murcha IAC	Brazil	CCSM	Folha Murcha IAC 474
IPR Folha Murcha	Brazil	IDR^2^	IPR 172 Folha Murcha

1 CCSM – Centro de Citricultura “Sylvio Moreira” Cordeirópolis, SP, Brazil.

2 IDR-Paraná – Instituto de Desenvolvimento Rural do Paraná - IAPAR/EMATER, Londrina, Paraná, Brazil.

### Orchard Management

Citrus tree management was based on the recommendations for the state of Paraná, Brazil (IAPAR, 1992; Nunes et al., 2010). Fertilizations were performed based on soil analysis. Weeds were managed with periodic mowing using an ecological rotary mower and herbicides sprays, between and within rows, respectively. Trees were irrigated with a localized drip irrigation system. The amount of water supplied to the trees was determined according to the crop evapotranspiration (*ETc*), which is determined by the crop coefficient procedure whereby the effect of the various weather conditions is incorporated into the reference crop evapotranspiration (*ETo*), and the crop characteristics into the single crop coefficient (*Kc*; Allen et al., 1998):


ETc=ETo×Kc,

where ETc = crop evapotranspiration, *ETo* = reference crop evapotranspiration, and *Kc* = single crop coefficient.

Disease and insect pest management included monthly preventive sprays to control citrus canker and leafminer (Behlau et al., 2010, 2021), and two-monthly insecticide sprays to control the ACP (*D. citri* Kuwayama) from fruit set to fruit maturation (Nunes et al., 2010). Top and hedge pruning were not performed in order to evaluate the natural tree growth.

### Vegetative Growth Measurements

The vegetative growth of the trees was assessed in early-spring of 2019, when the trees were seven-year-old. Tree canopy volume (CV) was determined based on tree height (TH) and canopy diameter (CD) measured with a graduate pole according to Mendel (1956):


$$\mathrm{CV}=\frac{2}{3}\times \pi \times CR^{2}\times TH,$$

where CV = canopy volume (m^3^); *CR* = canopy radius (m); and *TH* = tree height (m).

Trunk circumference was measured 10 cm above and 10 cm below the graft union using a cloth measuring tape and converted to diameter. The trunk index was calculated based on the relationship between the trunk diameter above and below the graft union.

### Fruit Yield and Production Efficiency

Fruit yield was evaluated annually from 2016 to 2021 in November of each year, about 15 months after the main bloom from the tree innermost trees. Cumulative yield was determined after the annual harvests. The yield efficiency was determined based on the relationship between the fruit yield average (kg per tree) and canopy volume (m^3^ per tree) assessed in 2019. The result was expressed in kilograms per cubic meter (kg.m^−3^) of tree canopy. The alternate bearing index was determined according to Pearce and Dobersek-Urbanc (1967):


$$\mathrm{ABI}=\frac{1}{n-1}\times \left\{\frac{\left|a_{2}-a_{1}\right|}{a_{2}+a_{1}}+\frac{\left|a_{3}-a_{2}\right|}{a_{3}+a_{2}}+\cdots +\frac{\left|a_{n}-a_{n-1}\right|}{a_{n}+a_{n-1}}\right\},$$

where ABI = alternate bearing index; *n* = number of years; and *a_1_, a_2_*, *… a_(*n*)_*, *a_(*n*-1)_* = yields of the corresponding year.

### Fruit Quality Evaluations

Fruit quality attributes were determined based on 10-fruit samples collected from the three innermost trees of each block. Fruit was randomly collected at 1–2 m tree height in October–November of each year from 2019 to 2021, before the annual harvests, with the averages for the evaluation period being presented. Fruit length and diameter were measured with a digital Vernier caliper (Mitutoyo, ABS, Kawasaki, Japan), weighed and classified according to the fresh citrus standards (CEAGESP, 2011). The fruit shape index was calculated based on the relationship between fruit length and diameter.

Fruit and juice colors were measured using a portable chroma meter (Minolta CR-400, Konica Minolta, Tokyo, Japan) and the *CIELab* color system. The device was calibrated before color assessments with a white tile. Fruit color was measured by readings taken at four equidistant points in the equatorial circumference of the fruit. Juice color was determined for each sample by readings taken through a 10 ml cuvette filled with juice. The citrus color index (CCI) was calculated based on previous report (Jiménez-Cuesta et al., 1981):


$$\mathrm{CCI}=\frac{1000\times a^{*}}{L^{*}\times b^{*}},$$

where CCI = citrus color index, a* = red-green color value, b* = yellow-blue color value, L* = lightness.

The CCI is a comprehensive indicator for color impression. The CCI values below −7 represents green; values within −7 and 0 indicate shades of light green passing through the yellowish green color; values within 0 and 7 represent the pale yellow to orange color; and values above 7 indicate the orange color, rising in intensity as the CCI increases (Jiménez-Cuesta et al., 1981). After assessing fruit color, juice aliquots were sampled using a Croydon extractor (Croydon, Duque de Caxias, Brazil). Juice content (JC) was determined according to the following equation and expressed as percentage:


$$\mathrm{JC}=\frac{JW}{FW}\times 100,$$

where JC = juice content; *JW* = juice weight (g) and *FW* = fruit weight (g).

Total soluble solids (TSS) concentration was measured with a digital refractometer (Atago Co., Ltd., PAL-3, Tokyo, Japan) in 0.3 mL of undiluted juice. The results were expressed in °Brix units. Titratable acidity (TA) was determined in 25 mL juice and 0.1 N NaOH in a TitroLine easy titrator (Schott Instruments GmbH, Mainz, Germany), and expressed in grams of citric acid per 100 mL of juice (g.100 mL^−1^; AOAC, 2019). The maturity index (TSS.TA^−1^ ratio) was calculated to determine the fruit maturity. The technological index, which indicates the amount of TSS content per standard citrus box (total capacity of 40.8 kg), was calculated according to the equation proposed by Di Giorgi et al. (1990):


$$\mathrm{TI}=\frac{TSS\times JC\times 40.8}{10000},$$

where TI = technological index (kg TSS.box^−1^); *TSS* = total soluble solids (°Brix); and *JC* = juice content (%).

### Estimates for Planting Density and Yield

Based on tree size measures in 2019, the corresponding number of trees per hectare was estimated for all sweet orange selections included in this study, assuming a free spacing of 2.5 m between-rows (canopy diameter + 2.5 m) and 25% tree overlap in-rows (canopy diameter *×* 0.75; De Negri and Blasco, 1991). Fruit yield was estimated according to the theoretical number of trees per hectare and the average fruit yield per tree determined for the 2017–2021 cropping seasons, when all trees were bearing. The soluble solids yield was determined according to the estimated yield and expressed in tons of TSS per hectare (t TSS.ha^−1^):


$$\mathrm{TSS}\;\mathrm{Yield}=\frac{TSS\times JC\times YE}{10000},$$

where TSS Yield = total soluble solids yield (t TSS.ha^−1^); *TSS* = total soluble solids (°Brix); *JC* = juice content (%); and *YE* = yield estimation. Adapted from Di Giorgi et al. (1990).

### Sweet Orange Performance Index for Fresh Fruit Market and Juice Processing

To calculate the performance index for each evaluated sweet orange selection, the data were normalized using the equations proposed by Ramos et al. (2021):


$$N1=\left|\frac{\max-\min}{2}\right|,$$

$$N2=\left|\frac{N1\times 100}{\max}\right|,$$

$$N3=\left|\frac{N2\times V}{N1}\right|,$$

where *max* = maximum value of each variable; *min* = minimum value of each variable; and *V* = value of the variable.

Results were used to calculate the sweet orange performance index for the fresh fruit market (FFI – fresh fruit index) and for the juice processing (JPI – juice processing index) according to Ramos et al. (2021):


$$\mathrm{FFI}\;\mathrm{or}\;\mathrm{IPI}=\left|\frac{\left(A\times a\right)+\left(B\times b\right)+\cdots +\left(N\times n\right)}{\left(\max-\min\right)}\right|,$$

where *A*, *B*, …, *N* = correspond to the indices of each variable; *a*, *b*, …, *n* = relative importance attributed to each variable (%); max = maximum value of each variable; and min = minimum value of each variable (Caputo et al., 2012).

The scores of each variable were assigned according to their relative importance for the fresh fruit market as it follows: 30% for cumulative yield, 20% for TSS, 20% for fruit color, 10% for number of seeds, and 20% for fruit weight; and for juice processing: 30% for cumulative yield, 30% for TSS, 30% for juice content, and 10% for juice color (Ramos et al., 2021).

### Incidence of Fruit With Citrus Canker

**Figure 3:**
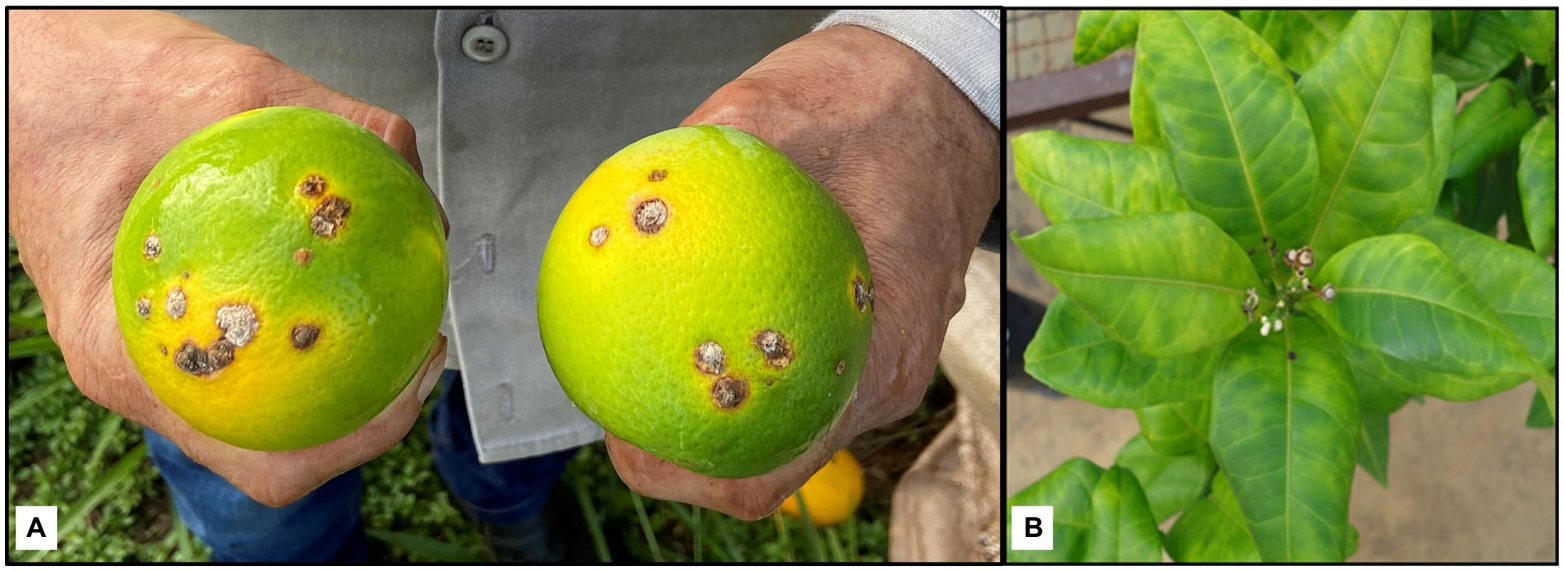
Citrus canker lesions in harvested fruit **(A)** and huanglongbing (HLB) symptoms in leaves **(B)** of sweet orange selections in Guairaçá, state of Paraná, Brazil.

Citrus canker incidence on fruits was evaluated at the annual harvests of 2020 and 2021 by visually assessing canker lesions on a 100-fruit sample per plot (Behlau et al., 2010). Diseased fruits usually present raised pinpoint spots that develop to brownish circular erumpent lesions, with a necrotic center surrounded by a chlorotic halo (Figure 3A). Fruit of each selection were collected from the three middle trees of each plot. The incidence of fruit with citrus canker at harvest was expressed in percentage (%).

### Incidence of Trees With HLB

Molecular analyses using the conventional polymerase chain reaction (PCR) technic were performed to determine the infection rate of the citrus trees by the huanglongbing (HLB) putative causal agent, ‘*Candidatus* Liberibacter asiaticus’ (*C*Las). Leaves, asymptomatic and HLB-symptomatic (Figure 3B), were sampled from the sweet orange trees in the late spring of the 2020 growth season and in the late fall and spring of 2021 growth season. Total genomic DNA was extracted (Murray and Thompson, 1980) from 10-leaf sample per tree of the five trees of each plot and subjected to PCR amplification with the primers sets A2/J5 (Hocquellet et al., 1999) and Oi1/Oi2c (Jagoueix et al., 1994; Supplementary Table 1). PCR reaction was performed in 20 μL of reaction mixture containing 0.1 μL of each primer (0.5 mM), 0.8 μL of dNTP (5 mM), 0.8 μL of MgCl_2_ (50 mM), 2.0 μL of buffer (10×), 1.0 μL of Taq DNA polymerase recombinant (5 U μL^−1^; Invitrogen, Carlsbad, United States), 14.2 μL of ultrapure water and 1 μL of total genomic DNA. PCR reactions were performed in a thermal cycler (Veriti^™^ 96-Well, Applied Biosystems^®^, Waltham, United States). For the A2/J5 primers, the thermal cycler was setup to 35 cycles at 92°C for 20 s, 62°C for 20 s, and 72°C for 45 s (Hocquellet et al., 1999); while for the Oi1/Oi2c primers, the device was configurated to 35 cycles at 92°C for 40 s, and 72°C for 90 s (Jagoueix et al., 1994). After amplification, one aliquot of each PCR reaction mixture was electrophoresed on 1.0% (w/v) agarose gel and visualized with SYBR^™^ Gold Nucleic Acid Gel Stain (ThermoFisher Scientific, Carlsbad, United States). The gel was visualized through a photo documenter (L-PIX EX, Loccus do Brasil Ltd., Cotia, Brazil) under ultraviolet (UV) light.

### Data Analyses

The data were analyzed according to the experimental design, tested for normal distribution and homogeneity at *p* ≤ 0.05, and then submitted to ANOVA. The citrus canker and HLB incidences data were transformed to arcsin √(x/100) before ANOVA. Means were grouped using the Scott-Knott’s test at *p* ≤ 0.05. Incidence of fruit with citrus canker was analyzed in a randomized block design with a factorial arrangement (19 sweet orange selections × 2 years). Significant variables were taken together and submitted to the multivariate analyses using a mean value for each selection and variable, and a principal component analysis (PCA) was plotted. All data were processed in *R* v. 4.0.2 (The R Foundation for Statistical Computing, Vienna, Austria) using the packages *ExpDes*, *ggplot2*, and *Facto MineR* for graphics and visualization of the statistical data.

## Results

### Tree Growth Measurements

Significant differences (*p* ≤ 0.05) among the 19 late-season sweet orange selections were observed for the vegetative measurements, including tree height, canopy diameter, canopy volume, and rootstock-scion trunk diameters (Table 2). The Valencia selections Olinda, Frost, Chafeei Late, #121, Campbell 479, Whit’s Late, Berry and Late Fla. had the tallest trees at range of 4.13–4.50 m, while Natal IAC, Valencia Late 161 and IPR Folha Murcha trees were the shortest ones varying from 3.56 to 3.79 m. Furthermore, trees of Olinda Valencia, Frost Valencia, Valencia #121 and Valencia Mutação exhibited the largest canopy diameters, differing from Campbell 479 Valencia, Natal IAC, Natal Murcha, Folha Murcha IAC and IPR Folha Murcha trees. Whereas Olinda Valencia trees showed the greatest canopy volume, with an average of 49 m^3^ per tree, and contrasted significantly with Natal AC, Natal Murcha, Folha Murcha IAC and IPR Folha Murcha trees, which developed a canopy volume smaller than 30 m^3^.

**Table 2:** Tree size of 19 late-season sweet orange selections grafted onto Rangpur lime rootstock and determined in the 2019 season. Guairaçá, state of Paraná, Brazil.

Sweet orange selection	Tree height (m)	Canopy diameter (m)	Canopy volume (m^3^)	Rootstock trunk diameter^1^ [Table-fn tfn3] (cm)	Scion trunk diameter^1^ (cm)	Trunk diameter index^2^
Whit’s Late Valencia	4.18 a^3^	4.12 b	37.2 c	17.8 b	15.6 b	0.88 c
Cutter Valencia	4.03 b	3.96 c	33.3 d	17.2 b	14.5 c	0.86 d
Berry Valencia	4.17 a	4.00 c	35.1 d	18.6 a	15.4 b	0.83 d
Frost Valencia	4.40 a	4.39 a	44.3 b	19.1 a	17.0 a	0.89 c
Valencia Mutação	4.08 b	4.38 a	41.0 c	15.4 c	13.2 d	0.85 d
Valencia IAC	3.89 b	3.87 c	30.7 d	16.4 c	14.5 c	0.89 c
Olinda Valencia	4.50 a	4.56 a	49.3 a	19.1 a	15.8 b	0.83 d
Chafeei Late Valencia	4.30 a	4.22 b	40.1 c	18.4 a	16.7 a	0.91 c
Campbell 479 Valencia	4.24 a	3.72 d	30.7 d	16.7 c	14.5 c	0.87 c
Campbell 294 Valencia	4.03 b	3.89 c	31.9 d	17.1 b	13.5 d	0.79 d
Valencia Late 161	3.79 c	3.89 c	30.0 d	16.5 c	15.4 b	0.93 b
Valencia Late Fla.	4.13 a	3.83 c	31.7 d	17.4 b	15.5 b	0.89 c
Valencia #121	4.27 a	4.39 a	43.1 b	18.1 b	15.7 b	0.87 c
Charmute de Brotas	3.94 b	4.10 b	34.6 d	16.2 c	14.3 c	0.88 c
Natal África do Sul	3.98 b	4.08 b	34.7 d	16.2 c	14.5 c	0.89 c
Natal IAC	3.56 c	3.74 d	26.2 e	15.9 c	13.9 c	0.87 c
Natal Murcha	3.91 b	3.65 d	27.3 e	14.9 d	13.6 d	0.91 c
Folha Murcha IAC	3.95 b	3.56 d	26.2 e	14.1 d	13.7 d	0.97 a
IPR Folha Murcha	3.81 c	3.68 d	27.1 e	13.2 d	12.2 e	0.92 b
CV (%)	3.88	3.66	8.28	5.09	4.95	3.04
*F* value	6.34^***^	11.53^***^	15.95^***^	10.59^***^	8.54^***^	7.02^***^

1 Trunk diameters were based on trunk circumference measurements, 10 cm above and 10 cm below the graft union.

2 Expressed as the ratio between scion and rootstock trunk diameters.

3 Means followed by the same letter in the column belong to the same group according to the Scott-Knott’s test. Significance level: ^***^p ≤ 0.001.

The rootstock trunk diameters of the Valencia selections Olinda, Frost, Berry, and Chafeei Late were significantly larger (18.4–19.1 cm) than those observed for Natal Murcha, Folha Murcha IAC and IPR Folha Murcha (13.2–15.9 cm; Table 2). Frost and Chafeei Late Valencia trees scored the largest scion trunk diameters among the evaluated sweet oranges included in the study (~17 cm), while IPR Folha Murcha had the smallest diameter (12 cm). Similarly, the scion-rootstock trunk diameter ratios, expressed as trunk diameter indices, were also variable among the evaluated selections. The highest trunk diameter index was observed for IPR Folha Murcha (0.97), which differed significatively from those of some Valencia selections including Campbell 294, Mutação, Olinda, Berry, and Cutter.

### Fruit Yield and Production Efficiency

The highest fruit yields was observed for Whit’s Late Valencia and Natal África do Sul over the evaluated period (Table 3). The annual yield average for these two selections were above 100 kg per tree (Table 3). In 2016, when trees were near 4 years old, all trees exhibited lower yields or even did not produce any fruit, as was the case of Olinda Valencia. However, a significant increase on fruit production was observed from 2018 onwards for most of the selections, as the case of Whit’s Late Valencia and Natal África do Sul, but decreased in 2021 due to intense drought occurred during this cropping season. Similarly, these selections had the highest cumulative yield after six consecutive years of fruit production, with 683 kg per tree for Whit’s Late Valencia and 646 kg per tree for Natal África do Sul (Table 3). The lowest alternate bearing indices were found for the Valencia selections Cutter, IAC, Campbell 479, Late Fla. and for Charmute de Brotas and Folha Murcha IAC, differing significantly from Olinda Valencia. Most of the evaluated selections grouped for the highest yield efficiency, except the Valencia selections Berry, Frost, Mutação, Olinda, Chafeei Late, Late 161 and #121 that exhibited poor yield efficiency.

**Table 3:** Yield performance of 19 late-season sweet orange selections grafted onto Rangpur lime rootstock from 2016 to 2021. Guairaçá, state of Paraná, Brazil.

Sweet orange selection	Yield (kg per tree)						Cumulative yield (kg)	Alternate bearing index	Yield efficiency (kg.m^−3^)^1^
	2016	2017	2018	2019	2020	2021			
Whit’s Late Valencia	47.1 b^2^	120.3 a	125.3 a	128.7 a	158.0 b	103.8 c	683.1 a	0.20 b	3.48 a
Cutter Valencia	64.1 a	89.8 b	118.4 a	123.7 a	115.3 c	98.0 c	609.3 b	0.10 c	3.30 a
Berry Valencia	49.5 b	58.2 c	73.4 b	67.6 b	145.5 b	86.5 d	480.3 c	0.22 b	2.45 b
Frost Valencia	40.4 b	59.6 c	106.8 a	84.5 b	104.8 c	102.5 c	498.7 c	0.20 b	2.08 b
Valencia Mutação	31.0 b	115.1 a	116.5 a	118.1 a	143.3 b	74.5 e	598.4 b	0.23 b	2.78 b
Valencia IAC	43.0 b	93.5 b	104.5 a	101.3 b	132.7 c	120.0 b	594.9 b	0.15 c	3.70 a
Olinda Valencia	–^3^	46.4 c	96.0 a	74.3 b	180.8 a	72.5 e	475.9 c	0.34 a	1.94 b
Chafeei Late Valencia	46.9 b	80.0 b	100.6 a	72.2 b	125.5 c	99.0 c	524.2 c	0.20 b	2.38 b
Campbell 479 Valencia	57.2 a	90.6 b	89.6 a	97.2 b	147.5 b	117.8 b	599.9 b	0.14 c	3.55 a
Campbell 294 Valencia	54.4 a	89.9 b	91.5 a	94.8 b	143.0 b	57.0 e	530.4 c	0.22 b	2.98 a
Valencia Late 161	28.3 b	52.9 c	66.2 b	80.2 b	101.0 c	64.0 e	392.6 c	0.22 b	2.42 b
Valencia Late Fla.	54.0 a	84.1 b	55.1 b	111.8 a	100.5 c	101.5 c	507.0 c	0.17 c	2.86 a
Valencia #121	45.7 b	41.5 c	83.1 b	80.8 b	152.0 b	144.0 a	547.2 c	0.20 b	2.33 b
Charmute de Brotas	71.3 a	74.1 c	106.0 a	104.5 a	146.0 b	87.1 d	588.7 b	0.14 c	3.00 a
Natal África do Sul	59.7 a	114.0 a	81.2 b	141.2 a	161.0 b	89.0 d	646.1 a	0.22 b	3.38 a
Natal IAC	58.5 a	83.4 b	75.1 b	79.2 b	109.8 c	65.8 e	471.8 c	0.14 c	3.15 a
Natal Murcha	34.5 b	78.7 b	66.3 b	101.8 a	124.3 c	98.3 c	503.8 c	0.19 b	3.46 a
Folha Murcha IAC	54.1 a	71.9 c	61.7 b	94.9 b	112.2 c	72.2 e	466.9 c	0.15 c	3.15 a
IPR Folha Murcha	45.0 b	66.8 c	78.4 b	81.0 b	126.5 c	79.8 d	477.5 c	0.21 b	3.23 a
CV (%)	19.90	16.72	21.52	24.03	12.66	13.20	7.80	24.17	11.35
*F* value	4.03^***^	8.51^***^	3.40^***^	2.38^*^	5.67^***^	9.88^***^	9.18^***^	3.77^***^	7.45^***^

1 Yield efficiency was based on the average yield from 2017 through 2021 and the canopy volume assessed in 2019.

2 Means followed by the same letter in the column belong to the same group according to the Scott-Knott’s test. Significance level: ^*^p ≤ 0.05; ^***^p ≤ 0.001.

3 No data for the respective year.

### Fruit Quality Evaluation

**Figure 4:**
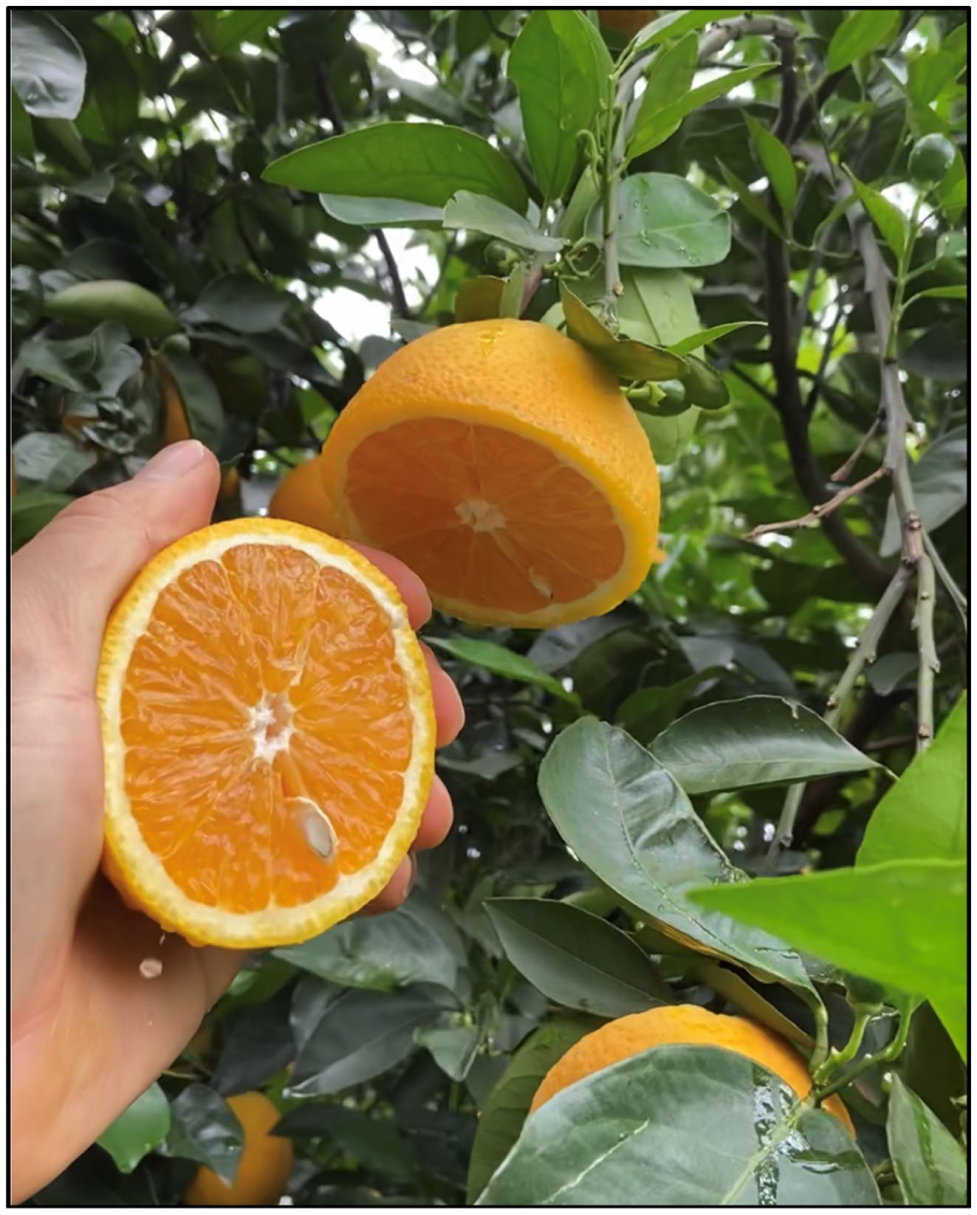
Fruit of nine-year-old Frost Valencia sweet orange tree grafted on Rangpur lime rootstock in Guairaçá, state of Paraná, Brazil.

Based on the average of three consecutive cropping seasons (2019–2021), significant differences were observed among the sweet orange selections for all fruit quality attributes assessed (Tables 4, 5). The largest fruit length (81.9 mm) was recorded for fruits of Folha Murcha IAC and some Valencia selections as Olinda, #121, Frost, Chafeei Late, Whit’s Late, and Late Fla. (Table 4). Similarly, Valencia #121 had the largest fruit diameter. Moreover, fruit produced by Folha Murcha IAC was oblong in shape, differing from all other selections, producing round-shaped fruits. Fruit weight ranged from 197 to 251 g, depending on the selection. The Valencia selections #121, Whit’s Late, Late 161, and Frost, and the Charmute de Brotas fruits were significant heavier than fruits of all other sweet oranges (Table 4). The number of seeds per fruit were low, one to five seeds per fruit, for all sweet orange selections. Olinda Valencia, Valencia Late 161 and Charmute de Brotas produced fruit with a much lower number of seeds than Campbell 479 Valencia. Regarding the fruit color, the fruits of all Valencia selections showed higher color index (Figure 4), within −0.04 and 1.34 CCI, than Natal África do Sul, Natal Murcha and Folha Murcha IAC (Table 4).

**Table 4:** Three-season average fruit quality of 19 late-season sweet oranges produced in Guairaçá, state of Paraná, Brazil, from 2019 to 2021.

Sweet orange selection	Fruit length FL (mm)	Fruit diameter FD (mm)	Fruit shape (FL.FD^−1^)	Fruit weight (g)	Number of seeds	Peel color (CCI)
Whit’s Late Valencia	78.9 a^1^	78.5 b	1.00 c	240 a	3 b	1.34 a
Cutter Valencia	77.8 b	78.5 b	0.99 c	222 b	3 b	0.10 a
Berry Valencia	76.8 b	76.2 b	1.01 c	211 b	3 b	0.89 a
Frost Valencia	79.7 a	79.3 b	1.00 c	231 a	2 c	0.90 a
Valencia Mutação	77.2 b	77.7 b	0.99 c	214 b	3 b	1.27 a
Valencia IAC	76.6 b	77.5 b	0.99 c	217 b	4 b	0.07 a
Olinda Valencia	80.2 a	77.1 b	1.04 b	212 b	1 d	0.81 a
Chafeei Late Valencia	79.6 a	79.6 b	1.00 c	216 b	4 b	0.15 a
Campbell 479 Valencia	75.5 b	76.6 b	0.99 c	209 b	5 a	0.78 a
Campbell 294 Valencia	75.8 b	78.3 b	0.97 c	216 b	4 b	0.01 a
Valencia Late 161	76.9 b	77.6 b	0.99 c	233 a	1 d	0.71 a
Valencia Late Fla.	78.7 a	77.9 b	1.01 c	211 b	4 b	0.91 a
Valencia #121	80.2 a	81.5 a	0.98 c	251 a	3 b	0.09 a
Charmute de Brotas	76.7 b	76.2 b	1.01 c	226 a	1 d	−0.04 a
Natal África do Sul	75.0 b	75.6 b	0.99 c	197 b	4 b	−0.53 b
Natal IAC	76.6 b	76.2 b	1.00 c	205 b	2 c	0.19 a
Natal Murcha	75.9 b	78.1 b	0.97 c	219 b	3 b	−1.76 b
Folha Murcha IAC	81.9 a	75.8 b	1.08 a	214 b	3 b	−1.76 b
IPR Folha Murcha	76.2 b	76.9 b	0.99 c	209 b	2 c	0.48 a
CV (%)	2.10	2.23	1.50	5.13	21.29	14.97
*F* value	4.11^***^	2.17^*^	8.12^***^	3.96^***^	9.42^***^	3.65^***^

1 Means followed by the same letter in the column belong to the same group according to the Scott-Knott’s test. Significance level: ^*^p ≤ 0.05; ^***^p ≤ 0.001.

**Table 5:** Three-season average juice quality of 19 late-season sweet oranges produced in Guairaçá, state of Paraná, Brazil, from 2019 to 2021.

Sweet orange selection	Juice color (CCI)	Juice content (%)	Total soluble solids TSS (°Brix)	Titratable acidity TA (g 100.mL^−1^)	Maturity index (TSS.TA^−1^)	Technological index (kg TSS.box^−1^)
Whit’s Late Valencia	−4.97 c^1^	44.9 a	10.1 b	1.08 a	9.4 e	1.86 a
Cutter Valencia	−2.70 a	43.7 a	10.3 b	0.79 c	13.1 c	1.83 a
Berry Valencia	−4.67 c	43.9 a	10.0 b	0.98 b	10.3 e	1.80 a
Frost Valencia	−3.53 b	39.0 c	10.4 a	1.06 a	9.9 e	1.66 b
Valencia Mutação	−3.43 b	44.2 a	10.2 b	0.75 d	13.5 c	1.83 a
Valencia IAC	−4.83 c	44.1 a	10.5 a	0.81 c	13.0 c	1.89 a
Olinda Valencia	−4.70 c	26.9 e	9.1 c	0.74 d	12.3 d	1.06 d
Chafeei Late Valencia	−4.23 c	44.6 a	10.1 b	0.93 b	11.0 d	1.84 a
Campbell 479 Valencia	−4.87 c	43.2 a	10.6 a	0.83 c	12.8 c	1.87 a
Campbell 294 Valencia	−3.60 b	41.0 b	9.9 b	0.72 d	13.7 c	1.65 b
Valencia Late 161	−3.70 b	41.6 b	10.7 a	0.79 c	13.6 c	1.81 a
Valencia Late Fla.	−4.40 c	42.9 a	10.9 a	0.98 b	11.2 d	1.91 a
Valencia #121	−4.77 c	42.0 b	10.5 a	0.90 b	11.7 d	1.80 a
Charmute de Brotas	−2.33 a	36.3 d	11.1 a	0.75 d	15.0 b	1.65 b
Natal África do Sul	−1.70 a	38.8 c	10.6 a	0.69 d	15.5 b	1.67 b
Natal IAC	−3.57 b	36.9 d	11.0 a	0.91 b	12.0 d	1.65 b
Natal Murcha	−2.33 a	39.0 c	11.0 a	0.66 d	16.8 a	1.76 a
Folha Murcha IAC	−2.13 a	37.7 d	9.7 b	0.73 d	13.3 c	1.44 c
IPR Folha Murcha	−3.13 b	39.5 c	10.9 a	0.71 d	15.3 b	1.76 a
CV (%)	15.82	2.25	3.78	6.47	6.36	5.22
*F* value	9.78^***^	66.70^***^	5.31^***^	16.62^***^	17.94^***^	14.41^***^

1 Means followed by the same letter in the column belong to the same group according to the Scott-Knott’s test. Significance level: ^***^p ≤ 0.001.

The color of the fresh-squeezed juice ranged from −4.97 up to −1.70 CCI among the studied sweet orange selections (Table 5). Cutter Valencia, Natal África do Sul, Folha Murcha IAC, Natal Murcha and Charmute de Brotas presented high standards for this juice attribute. Juice contents were higher for most of the Valencia selections (≥39%), except for Olinda that ranked below all assessed sweet oranges, with 27% (Table 5). The total soluble solid (TSS) content in the juices ranged from 9.1 to 11.1 °Brix among the selections. The lowest TSS was for the juice of Olinda Valencia (9.1 °Brix) while Frost Valencia, Valencia IAC, Campbell 479 Valencia, Valencia Late 161, Valencia Late Fla., Valencia #121, Charmute de Brotas, Natal África do Sul, Natal IAC, Natal Murcha and IPR Folha Murcha had the highest TSS concentration, above 10.4 °Brix (Table 5). The Valencia selections Whit’s Late and Frost had the highest level of juice acidity (titratable acidity – TA), with 1.08 and 1.06 g of citric acid per 100 mL of juice, respectively. On the other hand, all other selections showed acidity levels below 1.0 g.100 mL^−1^. The maturity index, i.e., TSS.TA^−1^ ratio, fluctuated from 9.4 to 16.8, depending on the sweet orange. Juice from Natal Murcha fruits showed the highest maturity index, while the ones from Valencia selections including Whit’s Late, Frost and Berry ranked at the bottom for this qualitative attribute. Most selections produced fruits of proper technological potential [technological index (TI) ≥ 1.70] to be processed by the juice industry. However, Olinda Valencia sweet orange exhibited the lowest TI performance (≤1.10), due basically to its low-quality juice.

### Estimates for Planting Density and Yield

The largest row and tree spacings were estimated for Olinda Valencia, Valencia #121, Frost Valencia and Valencia Mutação, while Campbell 479 Valencia, Folha Murcha IAC, Natal Murcha, IPR Folha Murcha and Natal IAC required the smallest spacing, maximizing tree density to a range from 572 up to 619 trees.ha^−1^ (Table 6). In contrast, the selections that demanded the largest tree and row spacings support no more than 415 and 444 trees.ha^−1^, which poses a restriction for their use in high-density plantings. Furthermore, Natal Murcha, Natal África do Sul and the Valencia selections Campbell 479, IAC, Whit’s Late and Cutter showed the highest estimation yield per area (t.ha^−1^), a favorable characteristic for high-density orchards. The estimates study also indicated that the sweet oranges with high TSS yield, as the Valencia selections Whit’s Late, IAC and Campbell 479, had their excellent performance due to their outstanding yield and juice quality (Tables 3, 6).

**Table 6:** Estimates^1^ of minimum row and tree spacing, maximum tree density, fruit yield, and TSS yield for 19 late-season sweet orange selections grafted onto Rangpur lime rootstock, based on field performance in Guairaçá, state of Paraná, Brazil.

Sweet orange selection	Row spacing (m)	Tree spacing (m)	Tree density (trees.ha^−1^)	Fruit yield (t.ha^−1^)	TSS yield (t TSS.ha^−1^)
Whit’s Late Valencia	3.09 b^2^	6.62 b	490 c	62.3 a	2.83 a
Cutter Valencia	2.97 c	6.46 c	522 b	56.9 a	2.57 b
Berry Valencia	3.01 c	6.51 c	512 c	43.9 c	1.93 c
Frost Valencia	3.29 a	6.88 a	443 d	40.6 c	1.64 c
Valencia Mutação	3.29 a	6.88 a	444 d	50.3 b	2.25 b
Valencia IAC	2.90 c	6.37 c	545 b	60.4 a	2.80 a
Olinda Valencia	3.42 a	7.06 a	415 d	39.4 c	0.96 d
Chafeei Late Valencia	3.16 b	6.72 b	471 c	44.9 c	2.02 c
Campbell 479 Valencia	2.79 d	6.22 d	578 a	62.8 a	2.89 a
Campbell 294 Valencia	2.91 c	6.39 c	537 b	51.1 b	2.07 c
Valencia Late 161	2.92 c	6.39 c	536 b	39.0 c	1.72 c
Valencia Late Fla.	2.87 c	6.33 c	551 b	49.9 b	2.34 b
Valencia #121	3.29 a	6.89 a	442 d	44.3 c	1.96 c
Charmute de Brotas	3.07 b	6.60 b	494 c	51.1 b	2.05 c
Natal África do Sul	3.06 b	6.58 b	497 c	58.3 a	2.39 b
Natal IAC	2.80 d	6.24 d	572 a	47.1 b	1.91 c
Natal Murcha	2.74 d	6.15 d	596 a	55.9 a	2.41 b
Folha Murcha IAC	2.67 d	6.06 d	619 a	51.1 b	1.87 c
IPR Folha Murcha	2.76 d	6.18 d	587 a	50.8 b	2.20 b
CV (%)	3.66	2.25	5.69	9.08	10.24
*F* value	11.53^***^	11.53^***^	11.85^***^	7.90^***^	13.38^***^

1 Estimates study was based on vegetative, yield, and fruit quality data of the evaluated selections; tree density and row/tree spacing projections were calculated according to De Negri and Blasco (1991) and used to estimate fruit yield and TSS yield.

2 Means followed by the same letter in the column belong to the same group according to the Scott-Knott’s test. Significance level: ^***^p ≤ 0.001.

### Sweet Orange Performance Index for Fresh Fruit Market and Juice Processing

The suitability for the fresh fruit market and for juice processing were evaluated for most sweet orange selections based on fruit quality and yield traits (Tables 3–5, 7), except by Olinda Valencia that did not attend the minimal standards (OECD, 2010; Berk, 2016) of the fresh fruit and industrial markets. All assessed late-season selections did not show any significant differences for the fresh fruit index (FFI), ranging from 1.04 (Frost Valencia) to 2.23 (Valencia IAC). In contrast, significant differences were observed among the sweet oranges for processing index (IPI; Table 7). Natal África do Sul and Charmute de Brotas scored the highest IPIs, an excellent performance for industrial processing. The lowest IPIs were observed for Valencia selections, including Whit’s Late, Berry, IAC, Chafeei Late, Campbell 479, Late 161, Late Fla. and #121, while the other selections showed intermediate indices for this attribute.

**Table 7:** Fresh fruit and industrial processing indices of 18 late-season sweet orange selections grafted onto Rangpur lime rootstock under humid subtropical conditions in Guairaçá, state of Paraná, Brazil, from 2016 to 2021 cropping season average.

Sweet orange selection	Fresh fruit index^1^	Industrial processing index^2^
Whit’s Late Valencia	1.30 a^3^	2.00 c
Cutter Valencia	1.31 a	4.39 b
Berry Valencia	1.28 a	2.06 c
Frost Valencia	1.04 a	3.83 b
Valencia Mutação	1.61 a	3.30 b
Valencia IAC	2.23 a	2.06 c
Chafeei Late Valencia	1.80 a	2.39 c
Campbell 479 Valencia	1.13 a	2.24 c
Campbell 294 Valencia	1.72 a	3.70 b
Valencia Late161	1.69 a	2.07 c
Valencia Late Fla.	1.38 a	2.40 c
Valencia #121	1.16 a	2.36 c
Charmute de Brotas	1.77 a	14.16 a
Natal África do Sul	1.35 a	16.55 a
Natal IAC	1.40 a	3.26 b
Natal Murcha	1.39 a	4.89 b
Folha Murcha IAC	1.40 a	3.30 b
IPR Folha Murcha	2.22 a	3.73 b
CV (%)	36.92	19.50
*F* value	1.12 ns	16.08^***^

1 Fresh fruit index was based on 30% cumulative yield, 20% TSS, 20% fruit color, 10% number of seeds and 20% fruit weight.

2 Industrial processing index was based on 30% cumulative yield, 30% TSS, 30% juice content and 10% juice color.

3 Means followed by the same letter in the column belong to the same group according to the Scott-Knott’s test. Significance level: ns, non-significant; ^***^p ≤ 0.001.

### Incidence of Citrus Canker and Huanglongbing

In regard to citrus canker incidence on harvested fruits, a highly significant (*p* ≤ 0.001) interaction was observed between the 19 late-season sweet orange selections and the two assessed harvest seasons, 2020 and 2021 (Table 8). As main effects, the highest incidence of citrus canker on fruits were found for those harvested from Valencia selections Frost and Late Fla. in both years, with more than 20% of affected fruits. These values were significantly higher (*p* ≤ 0.001) than those observed on fruits of IPR Folha Murcha, Natal IAC, Charmute de Brotas and Valencia selections Chafeei Late, Campbell 479, Campbell 294, Olinda, Mutação and Whit’s Late. Interestingly, Folha Murcha IAC had a substantial development of citrus canker on fruit from 2020 to 2021, showing an increase of tenfold. Similar trend was also observed in all other evaluated selections when comparing the 2020 and 2021 harvests (Table 8).

**Table 8:** Incidence of harvested fruit with citrus canker in 19 late-season sweet orange selections grafted onto Rangpur lime rootstock harvested in 2020 and 2021 in Guairaçá, state of Paraná, Brazil.

Source of variance	Year^1^	
	2020 harvest	2021 harvest
Sweet orange selection		
Whit’s Late Valencia	12.7 Bc^2^	17.7 Ae
Cutter Valencia	9.7 Bc	23.0 Ac
Berry Valencia	8.5 Bd	27.6 Ab
Frost Valencia	21.0 Aa	24.0 Ac
Valencia Mutação	8.0 Ad	10.5 Ag
Valencia IAC	10.0 Bc	27.7 Ab
Olinda Valencia	7.3 Bd	16.0 Ae
Chafeei Late Valencia	3.0 Be	17.7 Ae
Campbell 479 Valencia	6.3 Bd	14.3 Af
Campbell 294 Valencia	9.3 Bc	17.7 Ae
Valencia Late 161	10.3 Bc	21.3 Ad
Valencia Late Fla.	22.0 Ba	33.0 Aa
Valencia #121	17.7 Bb	29.3 Ab
Charmute de Brotas	7.0 Bd	16.5 Ae
Natal África do Sul	7.7 Bd	29.0 Ab
Natal IAC	6.7 Bd	25.7 Ac
Natal Murcha	15.3 Ab	14.5 Af
Folha Murcha IAC	3.3 Be	30.5 Aa
IPR Folha Murcha	4.0 Be	11.7 Ag
CV (%)	13.08	
Selection	37.0^***^	
Year	84.1^***^	
Selection × Year	17.82^***^	

1 All data were transformed to arcsin √(x/100) before submitted to ANOVA.

2 Means followed by same letter, capital case in the row and lowercase in the column, belong to the same group according to Scott-Knott’s test. Significance level: ^***^p ≤ 0.001.

**Figure 5:**
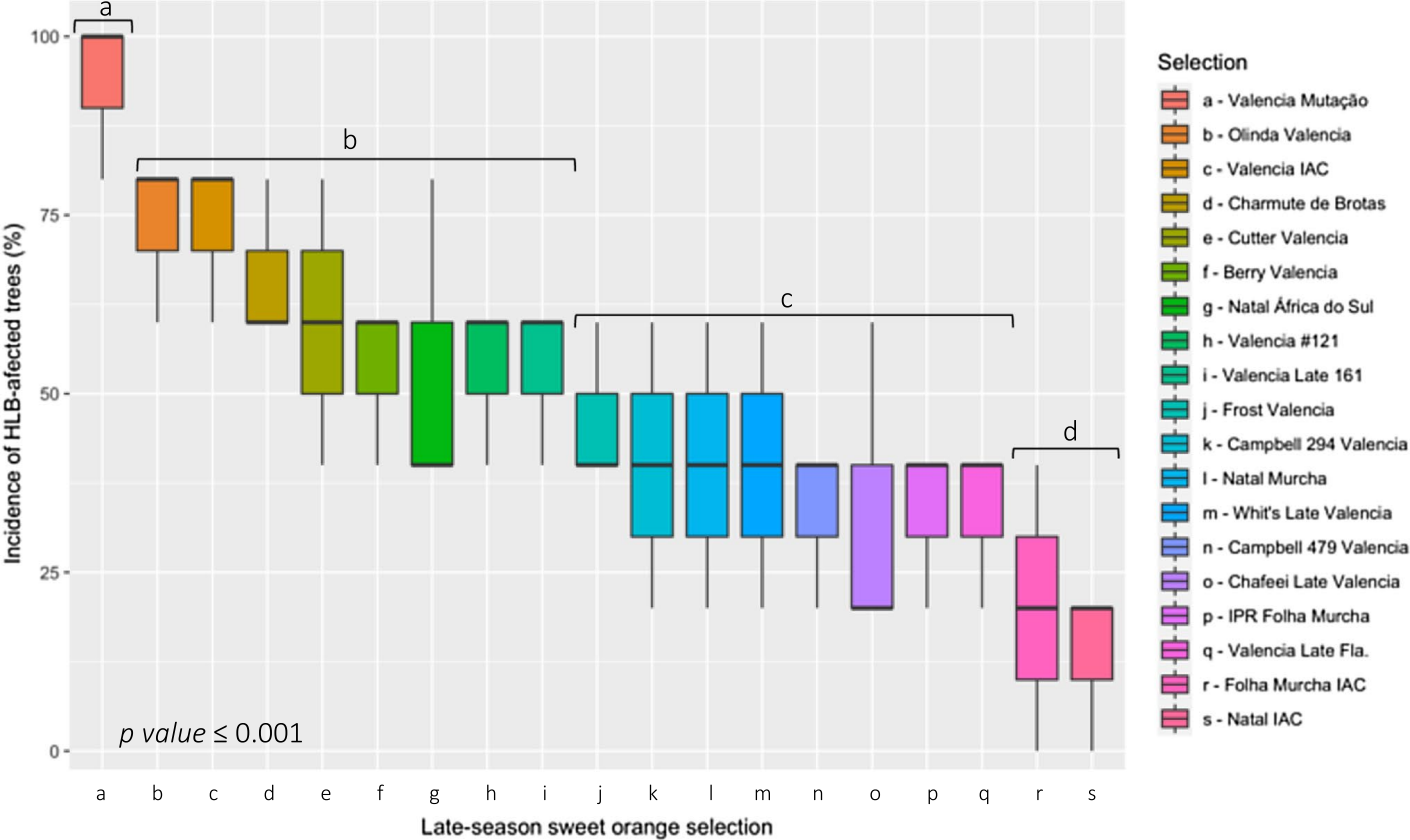
Cumulative incidence of huanglongbing (HLB)-affected trees in 19 late-season sweet orange selections grafted onto Rangpur lime rootstock after 9 years of planting in Guairaçá, state of Paraná, Brazil. HLB-affected trees were confirmed by the detection of ‘*Candidatus* Liberibacter asiaticus’ (*C*Las) infection in a polymerase chain reaction (cPCR) assay. All data were transformed to arcsin √(x/100) before submitted to ANOVA. Means followed by same letter in the bar, belong to the same group according to Scott-Knott’s test.

Significant differences were also observed within the assessed sweet orange selections for the cumulative incidence of HLB (Figure 5). The incidence of trees with PCR-confirmed HLB ranged from 13 to 93%. Nine years old trees of Valencia Mutação had the highest cumulative rate of *C*Las infection, differing significatively from all other selections (Figure 5). The lowest HLB incidence was observed for trees of Natal IAC and Folha Murcha IAC. All other selections showed intermediate levels of HLB incidence (Figure 5).

### Multivariate Analysis

**Figure 6:**
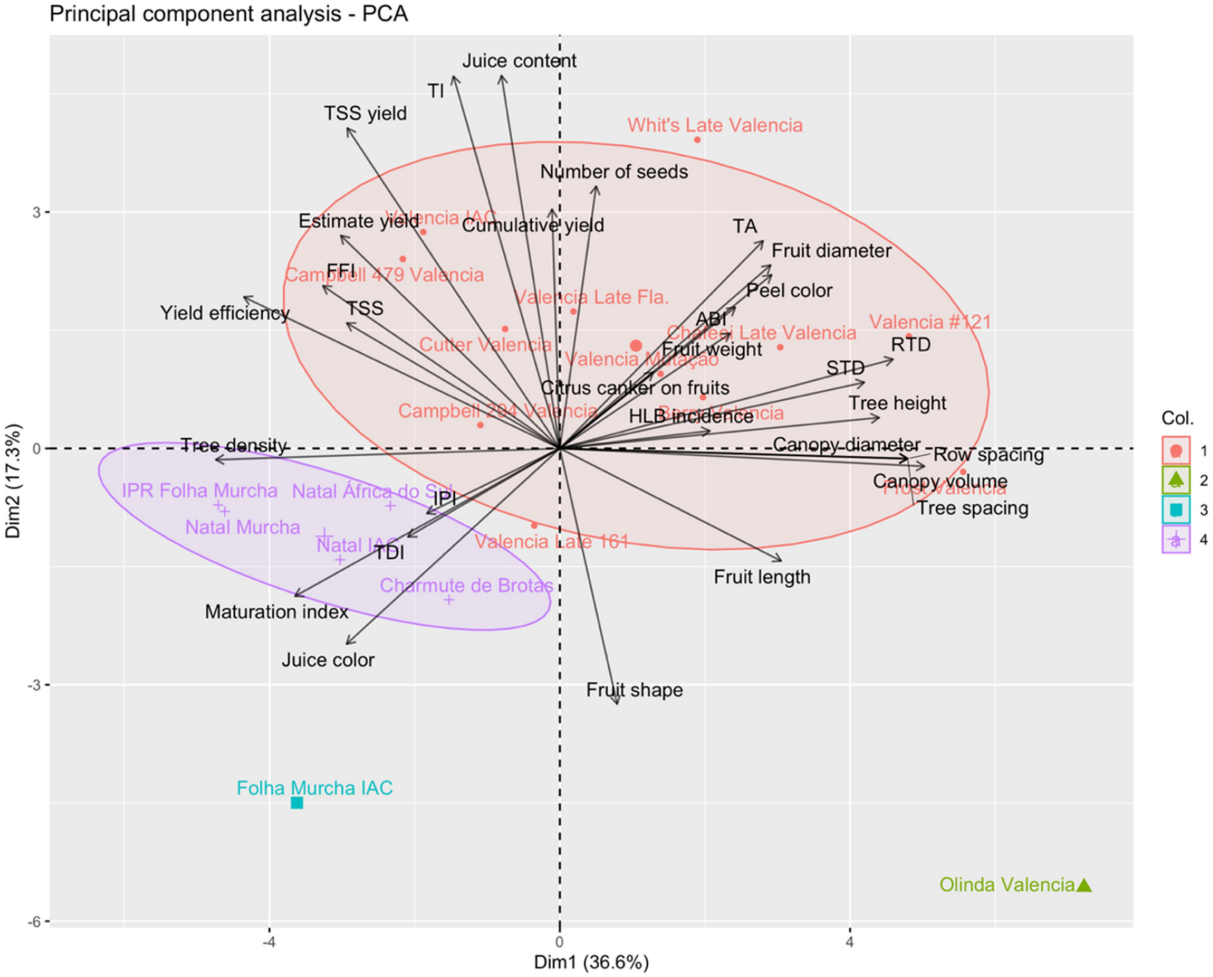
Principal component analysis (PCA) for tree size, yield, fruit quality, estimation of plant density and yield, incidence of citrus canker on fruits, and incidence of huanglongbing (HLB) in trees of 19 late-season sweet orange selections grafted onto Rangpur lime rootstock. The variables were arranged according to their principal component scores and the individuals (sweet orange selections) were grouped into four distinct clusters (Col.): 1, 2, 3, and 4. Variables: tree height (m); canopy volume (m^3^); canopy diameter (m); RTD – rootstock trunk diameter (cm); STD – scion trunk diameter (cm); TDI – trunk diameter index (STD.RTD^−1^); cumulative yield (kg per tree); yield efficiency (kg.m^−3^); ABI – alternate bearing index; row spacing (m); tree spacing (m); tree density (tree.ha^−1^); estimate yield (t.ha^−1^); TSS yield (t TSS.ha^−1^); fruit length (mm); fruit diameter (mm); fruit shape; fruit weight; number of seeds per fruit; peel color (CCI); juice color (CCI); juice content (%); TSS – total soluble solids (°Brix); TA – titratable acidity (g.100 mL^−1^); maturation index (TSS.TA^−1^); technological index (kg TSS.box^−1^); FFI – fresh fruit index; IPI – industrial processing index; incidence of citrus canker on fruits (%); and HLB incidence (%).

All assessed variables were submitted to the multivariate analysis, and a principal component analysis was built (Figure 6). The first two principal components accounted for 54% of the total variance in the dataset. Principal component 1 (Dim1) explained 37% of the variation while the principal component 2 (Dim2) represented 17%. Segregation among the 19 late-season sweet orange selections was observed when the two first principal components were projected. Four distinct groups (Col. 1, 2, 3, and 4) were formed based on the similarities of the sweet orange selections. The first group (Col. 1) comprised most of the Valencia selections including Whit’s Late, Cutter, Berry, Frost, Mutação, IAC, Chafeei Late, Campbell 294, Campbell 479, Late 161, Late Fla. and #121. These selections were recognized to be more productive and vigorous compared to the other sweet oranges (Figure 6). The vigorous tendency of Valencia resulted in lower tree density (Figure 6). Most Valencia trees produced fruits of better external quality including fruit size, weight, and color index. Some qualitative internal attributes were also remarkable within these sweet oranges as TSS, acidity and juice content. Exceptionally, Olinda Valencia formed a single group (Col. 2) far from all other accessions, mainly because this selection scored low for most assessed horticultural traits. Similarly, Folha Murcha IAC formed another single group (Col. 3), characterized to produce fruit of intense juice color but with a greenish peel color. Finally, Charmute de Brotas, Natal África do Sul, Natal IAC, Natal Murcha and IPR Folha Murcha grouped (Col. 4) together, because of their similarities on the evaluated traits. These selections were characterized to produce fruits of better juice quality based on maturity index and juice color, which favors their use for the processing, resulted from the highest IPIs.

## Discussion

Trees of 19 late-season sweet orange selections were evaluated for vegetative growth at the seven-year of age, when trees were well established in the field and stabilized in terms to vegetative growth and crop yield. Based on our findings, Olinda Valencia trees were significantly (*p* ≤ 0.001) the most vigorous among the tested sweet oranges. Further, this Valencia selection had the highest values for almost all vegetative parameters, except for the scion trunk diameter and the trunk diameter index. Olinda Valencia trees have also been recognized as vigorous in previous works (Hodgson et al., 1967; Castle and Baldwin, 2011; Torres and García, 2012; Simpson et al., 2014). Several other Valencia selections exhibited vigorous growth in our field trial, e.g., Frost, Berry, Whit’s Late, Chafeei Late, #121, Campbell 479 and Late Fla. These vigorous developments were also described in another study (Hodgson et al., 1967). On the other hand, Natal IAC, Natal Murcha, Folha Murcha IAC and IPR Folha Murcha selections had low vegetative growth in our field trial. These findings are in accordance with those previously reported by Stenzel et al. (2005) and Azevedo et al. (2015) for Folha Murcha and by Girardi et al. (2017) for Natal sweet orange.

Tree size plays an important role in the citrus orchard planning, field managements and other operations, including harvesting, pruning, irrigation and control of insect pests and diseases (Albrigo et al., 2019). Indeed, vegetative growth may determine tree density, depending as well on the tree architecture of each cultivar (Stuchi, 2005). Currently, the demand for small to medium-sized trees, which favors high-density plantings, have increased in commercial citrus-growing areas, particularly under endemic occurrence of HLB. It impacts the lifespan of the orchards changing the whole production system (Moreira et al., 2019; Bassanezi et al., 2020; Ferrarezi et al., 2020; Girardi et al., 2021; Carvalho et al., 2021b,c). Furthermore, high-density planting may assure higher yields at the young tree stage under the HLB pressure, favored by the increase of trees per area that optimizes the land use and improves the returns of the initial investments (Wheaton et al., 1991; Bassanezi et al., 2020). The smallest trees favored higher-density plantings and yields in our estimates study (Table 6). Among the smallest trees, we could include Folha Murcha IAC, IPR Folha Murcha, Natal IAC, Natal Murcha and Campbell 479 Valencia. On the other hand, the most vigorous selections allowed a limited number of trees per area and lower fruit yield, particularly Olinda Valencia (most vigorous; Table 6; Figure 6), which was also observed in our previous work for the mid-season sweet oranges Shamouti and Khalily White grown in the same region (Paula et al., 2022).

Although significant differences were apparent among the selections, all scions were graft-compatible with Rangpur lime. No overgrowth between scion and rootstock or tree decline was observed 9 years after planting. Our results are in agreement with previous reports indicating a broad affinity of Rangpur lime rootstock with several sweet orange cultivars and their relatives (Alves et al., 2021; Carvalho et al., 2021a, 2022; Cruz et al., 2021; Paula et al., 2022).

Fruit yield is essential for the evaluation of citrus germplasm. Genotypes that have higher yields and produce fruits of excellent quality in addition to tolerance or resistance to biotic and abiotic stresses are highly demanded by the citrus growers (Castle, 2010). Sweet orange trees usually start to produce fruits at two to 5 years of age (Di Giorgi et al., 1990; Castle et al., 2010). Currently, genotypes that show a tendency of early fruiting are very desirable due to the HLB pressure (Bové and Ayres, 2007; Spreen et al., 2007). Trees of Natal África do Sul and Whit’s Late Valencia had higher fruit production at the early stages, with four to 9 years of age. Here, the most vigorous trees had low fruit load in the first years, which includes the Valencia selections Olinda, #121 and Frost (Tables 2, 3). Moreover, Olinda trees did not show any fruit yield in 2016, starting to bear fruits later than all other selections, similar to the vigorous mid-season Shamouti and Khalily White trees mentioned in our early study assessing multiple sweet orange cultivars (Paula et al., 2022). Vigorous vegetative growth may limit fruit production in young trees (Carvalho et al., 2021a), as the photoassimilates may be directed to the vegetative growth at the expense of fruit growth and development (Agustí and Primo-Millo, 2020).

Fruit and juice quality are of major importance for the evaluation of sweet oranges. Quality standards of sweet oranges are regulated by different agencies around the world (OECD, 2010; CEAGESP, 2011). In our study, the fruit physical traits, including size, shape, weight, and color among the evaluated sweet orange selections were significantly different (*p* ≤ 0.05). Despite of the significant differences, all the sweet oranges produced fruits of large size (≤71 mm) and higher grade (A), according to the fresh fruit standards of the Brazilian fresh fruit market (CEAGESP, 2011).

The number of seeds per fruit is another important attribute to be considered in the evaluation of sweet oranges, as seedless fruits, or with a low number of seeds, are usually preferred by the fresh fruit market and juice processors (CEAGESP, 2011; Carvalho et al., 2019a). The number of seeds in the fruits can vary from zero to dozens (Ladaniya, 2008). In our study, all common sweet orange selections produced fruits with seeds, ranging from one to five seeds per fruit. However, they can still be considered commercially as seedless because they had less than eight seeds per fruit (Albrigo et al., 2019), particularly Valencia selections Late 161, Olinda and Frost, and Charmute de Brotas, Natal IAC and IPR Folha Murcha. Our findings agree with those reported in earlier studies for Valencia (Mesejo et al., 2007), Charmute de Brotas (Nascimento et al., 2005), Natal (Ladaniya, 2008; Couto et al., 2018) and Folha Murcha (Stuchi and Donadio, 2000).

The color development is not a reliable sign for maturity of sweet orange fruits (Berk, 2016; Gupta et al., 2021), though, this attribute may be critical for the fresh consumption (Ladaniya, 2008). The fresh market usually requires fruits with full coloration and without any peel damage (OECD, 2010; CEAGESP, 2011); while juice processors demand juice of intense and luminous coloration (Gama and Sylos, 2005). Juice color is not usually a concern with most mid- to late-season maturing sweet oranges (Castle and Baldwin, 2011), as they achieve better coloration than the early-season maturating cultivars (Fellers, 1990). In our study, Charmute de Brotas, Natal IAC, IPR Folha Murcha and all Valencia selections produced fruits of better peel color (Table 4; Figure 4). Regarding the juice color, Cutter Valencia, Charmute de Brotas, Natal África do Sul, Natal Murcha and Folha Murcha IAC fruits produced juices of better coloration, as the CCI were higher. It indicates an intense yellow coloration while all other fruit selections produced juice of yellowish green coloration (Table 5; Jiménez-Cuesta et al., 1981). The lowest juice content recorded in this fruit selections may have increased the concentration of colored compounds, as carotenoids, favoring for a deeper yellow coloration. Fruit of Valencia sweet orange is recognized to produce juice of high quality and intense coloration (Gama and Sylos, 2005). However, our results showed that Charmute de Brotas and some selections of Natal and Folha Murcha may also be potential alternatives for juice processing, as the color indices were significantly more yellow-intense than those observed for most of the Valencia selections (yellowish green).

Juice content may vary according to the region, due to climate and soil conditions, as well as to the rootstock, plant nutrition, irrigation, and tree age, among other factors (Castle et al., 2010; Berk, 2016; Albrigo et al., 2019). Sweet orange fruits should have at least 35% of juice content, according to the fresh market requirements (Ladaniya, 2008; OECD, 2010; CEAGESP, 2011), and 36–40% for juice processing (Chitarra and Chitarra, 2005; Lado et al., 2014; Albrigo et al., 2019). Fruits of almost all the sweet orange selections included in this study had juice content commercially acceptable, higher than the threshold established for the fresh fruit market and processing, except Olinda Valencia fruits that barely scored 26% of juice content. Despite of being planted in several important citrus-growing areas worldwide (Al-Jaleel and Zekri, 2002; Castle and Baldwin, 2011; Torres and García, 2012), Olinda Valencia selection did not show any reasonable horticultural performance under a humid subtropical climate. On the other hand, several other Valencia selections had high fruit juice content under the northwestern Paraná edaphoclimatic conditions, supporting the findings reported in previous studies for several sweet orange selections (Domingues et al., 2021; Paula et al., 2022).

Most of the sweet orange selections included in our study had TSS content higher than 10 °Brix, which is the minimum value required by the fresh fruit market in Brazil and in the United States (Arpaia and Kader, 2000; CEAGESP, 2011). The TSS contents were also above the international standard established by the Organization for Economic Co-operation and Development (OECD, 2010) and by the juice processing industry (Ladaniya, 2008), which is 8 °Brix. The lowest TSS content, 9.1°Brix, was for Olinda Valencia fruits. This value was lower than the one reported by Castle and Baldwin (2011) for this same selection grown in St. Cloud, FL, United States, for fruits from 5 years old trees, 12.7 °Brix. These variations may be related to tree age, soil-climate conditions, fertilization, and used rootstock (Albrigo et al., 2019). On the other hand, fruits of the Valencia selections Frost (Figure 4), IAC, Campbell 479, Late 161, Late Fla. and #121 as well as the Charmute de Brotas, Natal África do Sul, Natal IAC, Natal Murcha and IPR Folha Murcha grouped together for the highest TSS concentration (≥ 10 °Brix), excelling for this qualitative trait highly demanded by the fresh fruit market and industry.

The titratable acidity (TA) or acidity level, expressed as grams of citric acid per 100 mL of juice, ranged from 0.7 to 1.1 g.100 mL^−1^ in juices of the tested sweet orange selections. This acidity level is above the minimum demanded by the fresh market (Pozzan and Triboni, 2005) and within the range for juice processing, from 0.7 to 1.2 g.100 mL^−1^ (Berk, 2016). All sweet orange selections included in the study exhibited maturity index above the minimum of international standards for the fresh market, 6.5:1 (OECD, 2010). Moreover, the fruits of most sweet orange selections achieved the maturity level of 12, which is the threshold value demanded by the juice processors (Pozzan and Triboni, 2005), though they were bellow 16, considered as the maximum threshold value for human consumption (Jones and Cree, 1965).

Based on our findings, most sweet orange selections showed similar performance indices for the fresh fruit market. The exception was the Olinda Valencia that did not have a good performance in our field trial for the fresh and the industrial markets. For this reason, this selection was not included in the performance index estimates. On the other hand, all the other selections can be indicated for the fresh fruit market (Table 7). Significant differences were found among the selections for the performance index estimated for juice processing. Interestingly, Natal África do Sul and Charmute de Brotas had higher estimates, indicating a much better performance for the juice processing than some other tested selections.

Differences were also found among the sweet orange selections for the incidence of citrus canker on harvested fruits. Valencia Frost and Valencia Late Fla. had the highest incidence of the disease on fruits, differing from all other selections (Table 8). These two sweet oranges have been reported as very susceptible to citrus canker (Carvalho et al., 2015). Valencia Late Fla. showed a moderate resistance to citrus canker when trees were grown under protected conditions (Amaral et al., 2010), but were highly susceptible when cultivated under field conditions (Carvalho et al., 2015). IPR Folha Murcha, Natal Murcha, Charmute de Brotas and Valencia selections Chafeei Late, Campbell 479, Campbell 294, Olinda, Mutação and Whit’s Late had the lowest incidence of citrus canker on fruits agreeing with the results reported by Vargas et al. (2013) and Carvalho et al. (2015).

The incidence of citrus canker on harvested fruit progressed differently over the two seasons, 2020 and 2021 (Table 8). Most of these variations on canker incidence on fruits could be related to the environmental conditions for disease development, as higher temperature and intense rainfall are frequent during the rainy season in southern Brazil (Figure 1).

Citrus canker has become a serious problem for the Brazilian growers as the younger stage of fruit development coincides with the rainy season, from December to April (Amaral et al., 2010; Lanza et al., 2019), when the fruitlets are more susceptible to *X. citri* subsp. *citri* (*Xcc*) infection. In 2020, lower rainfall volume, 540 mm, was recorded during the rainy season comparing to 2021, 680 mm (Figure 1; Table 8). A higher rainfall volume in 2021 may have contributed to increase the incidence of the disease in fruits, favored by bacterial dispersion and further infection on fruits at the early stages of development. A longer rainy season during the early development of fruits may have promoted the progression and severity of the disease as previously noticed by Behlau et al. (2010). The highest canker incidence observed in the 2021 harvest weakly impacted on fruit yield according to the Pearson’s correlation coefficient (*r* = 0.18; value of *p* = 0.16), as a sightly reduction in production was observed comparing to the previous season (Tables 3, 8).

All tested sweet orange selections were susceptible to the infection by the *C*Las bacterium. Although there are no citrus cultivars immune to *C*Las (Rodrigues et al., 2020), significant differences (*p* ≤ 0.001) were found within the sweet orange selections in relation to the cumulative incidence of HLB (Figure 5). Natal IAC and Folha Murcha IAC had the lowest cumulative *C*Las-infected trees (Figure 5) over nine-year of field evaluation. It indicates an outstanding horticultural performance of these two selections under the humid subtropical climate, particularly Natal IAC, which was the last cultivar officially released for cultivation in the state of Paraná. Further, previous studies have demonstrated that the orchard arrangement may play an important role in the dispersion of the *C*Las vector, the ACP (Martini et al., 2015), and consequently in the incidence of HLB-infected trees in the orchard. Trees of different size at the same area have showed differences in the ACP population dynamics (Rodrigues et al., 2020). Larger trees may serve as a barrier preventing the ACP from landing on smaller trees. Furthermore, smaller trees may be less affected due to the flushing pattern of these trees (Rodrigues et al., 2020). The presence of smaller trees surrounded by larger ones may reduce their exposure to wind and sun, analogous to the effect of windbreaks (Martini et al., 2015). Windbreaks induce shading and temperature changes in orchards, which may reduce the tree flushing and consequently the attractiveness by the ACP, decreasing the incidence of HLB (Martini et al., 2015).

Folha Murcha IAC and IPR Folha Murcha have showed lower incidence of HLB-affected trees compared to Pera sweet orange under the same conditions of our field trial (Almeida et al., 2016). Lower HLB incidence in these two selections may be explained by the delayed bud break (Cantuarias-Avilés et al., 2011), the longer period to produce new shoots in early spring (Carvalho et al., 2021c), associated to the morphological characteristics of the curling leaves. Therefore, these phenological and morphological traits may favor the delay of new vegetative flushes, escaping from HLB bacterium transmission and consequent infection (Cifuentes-Arenas et al., 2018). As the ACP population typically peaks at late spring and early summer in Brazil and in some other important citrus-growing regions (Primo-Millo and Agustí, 2020; Carvalho et al., 2021c). Additionally, Folha Murcha sweet orange usually produces shoots of shorter lengths (Carvalho et al., 2021c), which limits the contact area for ACP feeding. Our results suggest that the variations in tree size, curled leaves, flushing pattern and time of flushes may have contributed to the response of these selections to the ACP low preference and, consequently, to the low HLB incidence in such sweet orange selections. Stover and McCollum (2011) also observed similar situation evaluating the HLB incidence in different citrus cultivars. Therefore, further investigations may be necessary to better determine the horticultural performance under homogeneous orchards of the late-season sweet orange selections that exhibited lower HLB incidence in our experimental trial.

Overall, the 19 assessed late-season sweet orange selections show significant differences for several horticultural traits, including vegetative growth, fruit yield and quality, row/tree spacing and yield estimates, performance for fresh fruit market and industrial processing, and incidences of citrus canker and cumulative HLB. IPR Folha Murcha, Natal IAC, Natal Murcha and Folha Murcha IAC had the smallest vegetative growth (Table 2; Figure 6), which favored the lowest tree/row spacings, and the highest tree density determined in the estimates study (Table 6). On the other hand, the majority of the Valencia selections had the most vigorous trees among the evaluated late-season sweet oranges, particularly Olinda that stayed alone from all other selections in the principal component analysis (Figure 6), requiring the largest spacings and the lowest tree density, similarly to Valencia #121 (Tables 2, 6). All the evaluated sweet orange selections were graft-compatible with the Rangpur lime rootstock, as no scion/rootstock overgrowth or tree decline were observed across 9 years of evaluation. Whit’s Late Valencia and Natal África do Sul were the most productive trees among the tested selections, with excelled yields over six cropping seasons (Table 3). Most late-season sweet orange selections produced fruits of excellent quality, except Olinda Valencia. Fruits of Valencia selections had the highest juice content with improved peel color (Tables 4, 5). Fruits of Charmute de Brotas, Natal África do Sul, Natal IAC, Natal Murcha, IPR Folha Murcha and the Valencia selections Frost, IAC, Campbell 479, Late 161, Late Fla., and #121 had juice of higher TSS contents. This favored an outstanding TSS.TA^−1^ ratio and juice color in the cases of Natal Murcha, Charmute de Brotas, Natal África do Sul, and IPR Folha Murcha (Table 5). All assessed late-season sweet oranges exhibited similar performances for the fresh fruit market, while Natal África do Sul and Charmute the Brotas were more indicated for industrial processing (Table 7). Furthermore, all evaluated selections exhibited different levels of citrus canker incidence on harvested fruits and HLB incidence in trees (Table 8; Figure 5). IPR Folha Murcha had the lowest incidence of canker on fruits while Folha Murcha IAC and Natal IAC expressed lower cumulative HLB incidence than all other tested selections, evidencing their excelled horticultural performance in the humid subtropical region.

## Conclusion

Based on our findings, Natal IAC, Folha Murcha IAC, IPR Folha Murcha, Natal Murcha, and the Valencia selections Campbell 479 and Late Fla. express better horticultural performance than other late-season selections under the humid subtropical climate. These selections have low HLB incidence, moderate incidence of citrus canker on fruits, outstanding yield efficiency, excellent fruit quality that attends the standards of the fresh fruit market and the juice processing industry, and low vegetative growth favoring higher tree density in new plantings. This optimizes the land use, field management and orchard operations, harvesting, yield, and faster return of the investments under an HLB endemic scenario. In contrast, the Valencia selections Olinda and Mutação are not recommended for planting in the Brazilian humid subtropical region or in regions with similar conditions, as these selections show poor horticultural performance. Together, our findings contribute to a more strategic and oriented recommendation of late-season sweet orange for the establishment of new plantings in the humid subtropical regions in the face of HLB pressure.

## Data Availability Statement

The original contributions presented in the study are included in the article/Supplementary Material, further inquiries can be directed to the corresponding author.

## Author Contributions

DC: investigation, data collection and formal data analysis, and writing – original draft. CN: supervision, writing – review and editing. MC: investigation, data collection and formal data analysis. TL: investigation, and data collection. FB and SC: conceptualization, writing – review and editing, funding acquisition, and resources. RL: supervision, conceptualization, investigation, writing – review and editing, funding acquisition, and resources. All authors contributed to the article and approved he submitted version.

## Funding

This study was partially funded by the Fundo de Defesa da Citricultura (Fundecitrus; Project No. 033038) and the Fundação de Amparo à Pesquisa do Estado de São Paulo (FAPESP; Process No. 2015/15779-9).

## Conflict of Interest

The authors declare that the research was conducted in the absence of any commercial or financial relationships that could be considered as a potential conflict of interest.

## Publisher’s Note

All claims expressed in this article are solely those of the authors and do not necessarily represent those of their affiliated organizations, or those of the publisher, the editors and the reviewers. Any product that may be evaluated in this article, or claim that may be made by its manufacturer, is not guaranteed or endorsed by the publisher.

## References

[ref1] Agustí M. Primo-Millo E. (2020). “Flowering and fruit set,” in The Genus Citrus. eds. Talon M. Caruso M. F. G. Jr. (Cambridge: Woodhead Publishing), 219–244.

[ref2] Albrigo L. G. Stelinski L. L. Timmer L. (2019). Citrus. Boston: CABI 324p

[ref3] Al-Jaleel A. Zekri M. (2002). Yield and fruit quality of ‘Olinda Valencia’ trees grown on nine rootstocks in Saudi Arabia. Proc. Fla. State Hort. Soc. 115, 17–22.

[ref4] Allen R. G. Pereira L. S. Raes D. Smith M. (1998). Crop Evapotranspiration: Guidelines for Computing Crop Water Requirements. Rome: Food and Agriculture Organization of the United Nations.

[ref01] Almeida E. P. D. Janeiro V. Guedes T. A. Mulati F. Carneiro J. W. P. Nunes W. M. D. C.. (2016). Modeling citrus huanglongbing data using a zero-inflated negative binomial distribution. Acta Sci. Agron. 38, 299–306. doi: 10.4025/actasciagron.v38i3.28689

[ref02] Alves M. N. Lopes S. A. Raiol-Junior L. L. Wulff N. A. Girardi E. A. Ollitrault P.. (2021). Resistance to ‘Candidatus Liberibacter asiaticus,’ the huanglongbing associated bacterium, in sexually and/or graft-compatible citrus relatives. Front. Plant Sci. 11:2166. doi: 10.3389/fpls.2020.617664 PMC7820388 33488659

[ref5] Amaral A. M. Carvalho S. A. Silva L. F. C. Machado M. A. (2010). Reaction of genotypes of citrus species and varieties to *Xanthomonas citri* subsp. *citri* under greenhouse conditions. J. Plant Pathol. 92, 519–524.

[ref6] AOAC-Association of Official Analytical Chemists (2019). Official Methods of Analysis of the AOAC International. Arlington: AOAC International.

[ref7] Arpaia M. Kader A. A. (2000). Recommendations for Maintaining Postharvest Quality. Davis: UC Davis.

[ref8] Auler P. A. M. Leite Júnior R. P. Tazima Z. H. Andrade P. F. S. A. (2014). Citricultura no Paraná. Citricultura Atual 99, 17–20.

[ref9] Azevedo F. A. Pacheco C. D. A. Schinor E. H. Carvalho S. A Conceição P. M. D. (2015). Produtividade de laranjeira Folha Murcha enxertada em limoeiro Cravo sob adensamento de plantio. Bragantia 74, 184–188. doi: 10.1590/1678-4499.0374

[ref10] Bassanezi R. B. Lopes S. A. Miranda M. P. Wulff N. A. Volpe H. X. L. Ayres A. J. (2020). Overview of citrus huanglongbing spread and management strategies in Brazil. Trop. Plant Pathol. 45, 251–264. doi: 10.1007/s40858-020-00343-y

[ref11] Behlau F. (2021). An overview of citrus canker in Brazil. Trop. Plant Pathol. 46, 1–12. doi: 10.1007/s40858-020-00377-2

[ref12] Behlau F. Belasque J. Graham J. H. Leite Júnior R. P. (2010). Effect of frequency of copper applications on control of citrus canker and the yield of young bearing sweet orange trees. Crop Prot. 29, 300–305. doi: 10.1016/j.cropro.2009.12.010

[ref13] Behlau F. Belasque Júnior J. Leite Júnior R. P. Filho A. B. Gottwald T. R. Graham J. H.. (2021). Relative contribution of windbreak, copper sprays, and leafminer control for citrus canker management and prevention of crop loss in sweet orange trees. Plant Dis. 105, 2097–2105. doi: 10.1094/PDIS-10-20-2153-RE, PMID: 33373290

[ref14] Berk Z. (2016). Citrus Fruit Processing. London: Elsevier Academic Press.

[ref15] Bové J. M. Ayres A. J. (2007). Etiology of three recent diseases of citrus in Sao Paulo state: sudden death, variegated chlorosis and huanglongbing. IUBMB Life 59, 346–354. doi: 10.1080/15216540701299326 17505974

[ref16] Cantuarias-Avilés T. Mourão Filho F. D. A. A. Stuchi E. S. Silva S. R. Espinoza-Nuñez E. (2011). Horticultural performance of ‘Folha Murcha’ sweet orange onto twelve rootstocks. Sci. Hortic. 129, 259–265. doi: 10.1016/j.scienta.2011.03.039

[ref17] Caputo M. M. Mourão Filho F. A. A. Silva R. S. Bremer Neto H. Couto H. T. Z. Stuchi E. S. (2012). Seleção de cultivares de laranja doce de maturação precoce por índices de desempenho. Pesqui. Agropecu. Bras. 47, 1669–1672. doi: 10.1590/S0100-204X2012001100015

[ref18] Carvalho D. U. Boakye D. A. Gast T. Leite Júnior R. P. Alferez F. (2021b). Determining seed viability during fruit maturation to improve seed production and availability of new Citrus rootstocks. Front. Plant Sci. 12:777078. doi: 10.3389/fpls.2021.777078, PMID: 34868177 PMC8641648

[ref19] Carvalho L. M. Carvalho H. W. Barros I. Martins C. R. Soares Filho W. D. S. Girardi E. A.. (2019b). New scion-rootstock combinations for diversification of sweet orange orchards in tropical hardsetting soils. Sci. Hortic. 243, 169–176. doi: 10.1016/j.scienta.2018.07.032

[ref20] Carvalho S. A. Nunes W. M. C. Belasque Júnior J. Machado M. A. Croce-Filho J. Bock C. H.. (2015). Comparison of resistance to Asiatic citrus canker among different genotypes of citrus in a long-term canker-resistance field screening experiment in Brazil. Plant Dis. 99, 207–218. doi: 10.1094/PDIS-04-14-0384-RE, PMID: 30699570

[ref21] Carvalho E. V. Cifuentes-Arenas J. C. Raiol-Junior L. L. Stuchi E. S. Girardi E. A. Lopes S. A. (2021c). Modeling seasonal flushing and shoot growth on different citrus scion-rootstock combinations. Sci. Hortic. 288:110358. doi: 10.1016/j.scienta.2021.110358

[ref22] Carvalho D. U. Cruz M. A. Colombo R. C. Tazima Z. H. Neves C. S. V. J. (2020). Fruit quality of ‘Salustiana’ seedless oranges during cold storage: effect of carnauba-based wax and rootstocks. J. Food Meas. Charact. 14, 3397–3407. doi: 10.1007/s11694-020-00583-1

[ref23] Carvalho S. A. Girardi E. A. Mourão Filho F. D. A. A. Ferrarezi R. S. Coletta Filho H. D. (2019a). Advances in citrus propagation in Brazil. Rev. Bras. Frutic. 41:e-422. doi: 10.1590/0100-29452019422

[ref24] Carvalho D. U. Neves C. S. V. J. Cruz M. A. Colombo R. C. Yada I. F. U. Leite Júnior R. P.. (2021a). Performance of ‘Salustiana’ sweet orange on different rootstocks under Brazilian subtropical conditions. Sci. Hortic. 287:110226. doi: 10.1016/j.scienta.2021.110226

[ref25] Carvalho D. U. Neves C. S. V. J. Cruz M. A. Yada I. F. U. Leite Júnior R. P. Tazima Z. H. (2022). Evaluation of multiple rootstocks for ‘Montenegrina’ mandarin in Londrina, Paraná, Brazil. Rev. Bras. Frutic. 44:e-817. doi: 10.1590/0100-29452022817

[ref26] Castle W. S. (2010). A career perspective on Citrus rootstocks, their development, and commercialization. HortScience 45, 11–15. doi: 10.21273/HORTSCI.45.1.11

[ref27] Castle W. S. Baldwin J. C. (2011). Young-tree performance of juvenile sweet orange scions on Swingle citrumelo rootstock. HortScience 46, 541–552. doi: 10.21273/HORTSCI.46.4.541

[ref28] Castle W. S. Baldwin J. C. Muraro R. P. Littell R. (2010). Performance of Valencia sweet orange trees on 12 rootstocks at two locations and an economic interpretation as a basis for rootstock selection. HortScience 45, 523–533. doi: 10.21273/HORTSCI.45.4.523

[ref29] CEAGESP - Companhia de Entrepostos e Armazéns Gerais de São Paulo (2011). Normas de classificação de citros de mesa. São Paulo: CEAGESP.

[ref30] Chitarra M. I. F. Chitarra A. B. (2005). Pós-colheita de frutas e hortaliças: fisiologia e manuseio. Lavras: UFLA.

[ref31] Cifuentes-Arenas J. C. De Goes A. De Miranda M. P. Beattie G. A. C. Lopes S. A. (2018). Citrus flush shoot ontogeny modulates biotic potential of *Diaphorina citri*. PLoS One 13:e0190563. doi: 10.1371/journal.pone.0190563, PMID: 29304052 PMC5755881

[ref32] Couto C. A. D. Souza E. R. B. D. Morgado C. M. A. Ogata T. Cunha L. C. (2018). *Citrus Sinensis* cultivars: alternatives for diversification of Brazilian orchards. Rev. Bras. Frutic. 40:e-097. doi: 10.1590/0100-29452018097

[ref33] Cruz M. A. Neves C. S. V. J. Carvalho D. U. Colombo R. C. Bai J. Yada I. F. U.. (2021). Five rootstocks for “emperor” mandarin Under subtropical climate in southern Brazil. Front. Plant Sci. 12:777871. doi: 10.3389/fpls.2021.777871, PMID: 34987531 PMC8722343

[ref34] De Negri J. D. Blasco E. E. A. (1991). “Planejamento e implantação de um pomar cítrico,” in Citricultura Brasileira. eds. Rodriguez O. Pompeu Júnior J. Viegas V. P. (Campinas: Fundação Cargill), 318–332.

[ref35] Di Giorgi F. Ide B. Y. Dib K. Marchi R. J. Triboni H. R. Wagner R. L. (1990). Contribuição ao estudo do comportamento de algumas variedades de citros e suas implicações agroindustriais. Citrus Res. Tech. 11, 567–612.

[ref36] Domingues A. R. Marcolini C. D. M. Gonçalves C. H. D. S. Resende J. T. V. D. Roberto S. R. Carlos E. F. (2021). Rootstocks genotypes impact on tree development and industrial properties of ‘Valencia’ sweet orange juice. Horticulturae 7:141. doi: 10.3390/horticulturae7060141

[ref37] FAO (Food Agricultural Organization) (2019). FAOSTAT: Production Crops. Available at: http://www.fao.org/faostat/en/#data (Accessed May 2, 2021).

[ref38] Fellers P. F. (1990). Florida’s juice standards for grades and their differences from United States Food and Drug Administration standards of identity. Proc. Fla. State Horti. Soc. 103, 260–265.

[ref39] Ferrarezi R. S. Jani A. D. James H. T. Gil C. Ritenour M. A. Wright A. L. (2020). Sweet orange orchard architecture design, fertilizer, and irrigation management strategies under huanglongbing-endemic conditions in the Indian River citrus district. HortScience 55, 2028–2036. doi: 10.21273/HORTSCI15390-20

[ref40] Gama J. J. T. Sylos C. M. (2005). Major carotenoid composition of Brazilian Valencia orange juice: identification and quantification by HPLC. Food Res. Int. 38, 899–903. doi: 10.1016/j.foodres.2005.03.008

[ref41] Girardi E. A. Cerqueira T. S. Cantuarias-Avilés T. E. Silva S. R. D. Stuchi E. S. (2017). Sunki mandarin and Swingle citrumelo as rootstocks for rain-fed cultivation of late-season sweet orange selections in northern São Paulo state, Brazil. Bragantia 76, 501–511. doi: 10.1590/1678-4499.2016.350

[ref42] Girardi E. A. Sola J. G. P. Scapin M. D. S. Moreira A. S. Bassanezi R. B. Ayres A. J.. (2021). The perfect match: adjusting high tree density to rootstock vigor for improving cropping and land use efficiency of sweet orange. Agronomy 11:2569. doi: 10.3390/agronomy11122569

[ref43] Gottwald T. R. (2010). Current epidemiological understanding of citrus huanglongbing. Annu. Rev. Phytopathol. 48, 119–139. doi: 10.1146/annurev-phyto-073009-114418, PMID: 20415578

[ref44] Gupta A. K. Pathak U. Tongbram T. Medhi M. Terdwongworakul A. Magwaza L. S.. (2021). Emerging approaches to determine maturity of citrus fruit. Crit. Rev. Food Sci. Nutr., 61, 1–22. doi: 10.1080/10408398.2021.1883547, PMID: 33583257

[ref45] Hocquellet A. Toorawa P. Bove J. M. Garnier M. (1999). Detection and identification of the two *Candidatus* Liberibacter species associated with citrus Huanglongbing by PCR amplification of ribossomal protein genes of the β operon. Mol. Cell. Probes 13, 373–379. doi: 10.1006/mcpr.1999.0263, PMID: 10508559

[ref46] Hodgson R. W. Reuther W. Webber H. J. Batchelor L. D. (1967). The Citrus Industry. Berkeley: University of California Press.

[ref47] IAPAR- Instituto Agronômico do Paraná (1992). A Citricultura no Paraná. Londrina: IAPAR (IAPAR Circular, 72).

[ref48] IBGE- Instituto Brasileiro de Geografia e Estatística (2020). Levantamento Sistemático da Produção Agrícola 2020. Available at: https://www.ibge.gov.br/estatisticas/economicas/agricultura-e-pecuaria/9201-levantamento-sistematico-da-producao-agricola.html?=&t=o-que-e. (Accessed December 3, 2021).

[ref49] IDR-Paraná (Instituto de Desenvolvimento Rural do Paraná) (2021). Estações meteorológicas. Available at: http://www.idrparana.pr.gov.br/Pagina/Dados-Meteorologicos-Historicos-e-Atuais (Accessed December 2, 2021).

[ref50] Jagoueix S. Bové J. M. Garnier M. (1994). The phloem-limited bacterium of greening disease of citrus is a member of the alpha subdivision of the Proteobacteria. Int. J. Syst. Bact. 44, 379–386. doi: 10.1099/00207713-44-3-379, PMID: 7520729

[ref51] Jiménez-Cuesta M. Cuquerella J. Martinez-Javaga J. M. (1981). Determination of a color index for citrus fruit degreening. Proc. Int. Soc. Citricul. 2, 750–753.

[ref52] Jones W. W. Cree C. B. (1965). Environment factors related to fruiting of Washington navel orange over a 38-year period. Proc. Am. Soc. Horti. Sci. 86, 267–271.

[ref53] Ladaniya M.S. (2008). Citrus Fruit: Biology, Technology and Evaluation. Goa, India: ICAR Research Complex for Goa.

[ref54] Lado J. Rodrigo J. M. Zacarias L. (2014). Maturity indicators and citrus fruit quality. Stewart Postharvest Rev. 2, 1–6.

[ref55] Lanza F. E. Marti W. Silva Júnior G. J. Behlau F. (2019). Characteristics of citrus canker lesions associated with premature drop of sweet orange fruit. Phytopathology 109, 44–51. doi: 10.1094/PHYTO-04-18-0114-R 29947244

[ref56] Larach J. O. I. Cardoso A. Carvalho A. P. Hochmüller D. P. Martins J. S. Rauen M. D. J.. (1984). Levantamento de Reconhecimento dos Solos do Estado do Paraná. Recife: Embrapa Solos.

[ref57] Leite Júnior R. P. Mohan S. K. (1990). Integrated management of the citrus bacterial canker disease caused by *Xanthomonas citri* subsp. *citri* in the state of Paraná, Brazil. Crop Prot. 9, 3–7. doi: 10.1016/0261-2194(90)90038-9

[ref58] Martini X. Pelz-Stelinski K. S. Stelinski L. L. (2015). Absence of windbreaks and replanting citrus in solid sets increase density of Asian citrus psyllid populations. Agric. Ecosyst. Environ. 212, 168–174. doi: 10.1016/j.agee.2015.06.027

[ref59] Mendel K. (1956). Rootstock-scion relationships in Shamouti trees on light soil. Ktavim 6, 35–60.

[ref60] Mesejo C. Martínez-Fuentes A. Reig C. Agustí M. (2007). The effective pollination period in ‘Clemenules’ mandarin, ‘Owari’ Satsuma mandarin and ‘Valencia’ sweet orange. Plant Sci. 173, 223–230. doi: 10.1016/j.plantsci.2007.05.009

[ref61] Moreira A. S. Stuchi E. S. Silva P. R. Bassanezi R. B. Girardi E. A. Laranjeira F. F. (2019). Could tree density play a role in managing Citrus Huanglongbing epidemics? Trop. Plant Pathol. 44, 268–274. doi: 10.1007/s40858-019-00284-1

[ref03] Murray M. G. Thompson W. F. (1980). Rapid isolation of high molecular weight plant DNA. Nucleic Acids Res. 8, 4321–4325. doi: 10.1093/nar/8.19.4321 7433111 PMC324241

[ref62] Nascimento L. M. Pompeu Júnior J. De Negri J. D. Zara F. Chignolli R. (2005). Laranja ‘Charmute de Brotas’: promissora variedade tardia. Citrus R&T 26, 69–75.

[ref63] Nunes W. M. C. Souza E. B. Leite Júnior R. P. Salvador C. A. Rinaldi D. A. Croce Filho J.. (2010). Plan of action for the control of huanglongbing in the Paraná state, Brazil. Citrus R&T 31, 169–177. doi: 10.5935/2236-3122.20100017

[ref64] Nunes W. M. C. Zanutto C. A. Rinaldi D. A. M. F. C. Croce Filho J. Azevedo M. L. Leite Júnior R. P.. (2007). Primeira constatação de huanglongbing em pomar comercial de citros no Estado do Paraná. Fitopatol. Bras. 32:1094

[ref65] OECD-Organization for Economic Co-operation and Development. (2010). Citrus Fruits, International Standards for fruit and Vegetables. Paris: OECD Publishing.

[ref66] Paula M. C. M. S. Carvalho D. U. Cruz M. A. Longhi T. V. Tazima Z. H. Behlau F.. (2022). Agronomic performance of sweet Orange genotypes under the Brazilian humid subtropical climate. Horticulturae 8:254. doi: 10.3390/horticulturae8030254

[ref67] Paula L. B. Lin H. Stuchi E. S. Francisco C. S. Safady N. G. Coletta-Filho H. D. (2019). Genetic diversity of ‘*Candidatus* Liberibacter asiaticus’ in Brazil analyzed in different geographic regions and citrus varieties. Eur. J. Plant Pathol. 154, 863–872. doi: 10.1007/s10658-019-01695-1

[ref68] Pearce S. C. Dobersek-Urbanc S. (1967). The measurement of irregularity in growth and cropping. J. Hortic. Sci. 42, 295–305. doi: 10.1080/00221589.1967.11514216

[ref69] Pozzan M. Triboni H. R. (2005). “Colheita e qualidade do fruto,” in Citros. eds. Mattos Júnior D. Negri J. D. Pio R. M. Júnior J. P. (Campinas: IAC/FUNDAG), 801–822.

[ref70] Primo-Millo E. Agustí M. (2020). “Vegetative growth,” in The Genus Citrus. eds. Talon M. Caruso M. Gmitter F. G. Jr. (Cambridge: Woodhead Publishing), 193–217.

[ref71] Ramos Y. C. Fadel A. L. Neto H. B. Caputo M. M. Stuchi E. S. Mourão Filho F. D. A. A. (2021). Mid-season sweet oranges for fresh and processing markets in Brazil. Exp. Agric. 57, 15–32. doi: 10.1017/S0014479721000016

[ref72] Rodrigues J. D. B. Moreira A. S. Stuchi E. S. Bassanezi R. B. Laranjeira F. F. Girardi E. A. (2020). Huanglongbing incidence, canopy volume, and sprouting dynamics of ‘Valencia’ sweet orange grafted onto 16 rootstocks. Trop. Plant Pathol. 45, 611–619. doi: 10.1007/s40858-020-00385-2

[ref73] Sauer A. V. Zanutto C. A. Nocchi P. T. R. Machado M. A. Bock C. H. Nunes W. M. (2015). Seasonal variation in populations of ‘*Candidatus* Liberibacter asiaticus’ in citrus trees in Paraná state. Brazil. *Plant Dis.* 99, 1125–1132. doi: 10.1094/PDIS-09-14-0926-RE, PMID: 30695933

[ref74] Simpson C. R. Nelson S. D. Melgar J. C. Jifon J. King S. R. Schuster G.. (2014). Growth response of grafted and ungrafted citrus trees to saline irrigation. Sci. Hortic. 169, 199–205. doi: 10.1016/j.scienta.2014.02.020

[ref75] Spreen T. H. Brown M. G. Muraro R. P. (2007). The projected impact of citrus greening in Sao Paulo and Florida on processed orange production and price. Proc. Fla. State Hort. Soc. 120, 132–135.

[ref76] Spreen T. H. Gao Z. Fernandes Junior W. Zansler M. L. (2020). “Global economics and marketing of citrus Products,” in The Genus Citrus. eds. Talon M. Caruso M. Gmitter F. G. Jr. (Cambridge: Woodhead Publishing), 471–493.

[ref77] Stenzel N. M. C. Neves C. S. V. J. Scholz M. B. D. S. Gomes J. C. (2005). Comportamento da laranjeira ‘Folha-Murcha’ em sete portas-enxerto no noroeste do Paraná. Rev. Bras. Frutic. 27, 408–411. doi: 10.1590/S0100-29452005000300017

[ref78] Stover E. D. McCollum G. (2011). Incidence and severity of huanglongbing and *Candidatus* Liberibacter asiaticus titer among field-infected citrus cultivars. HortScience 46, 1344–1348. doi: 10.21273/HORTSCI.46.10.1344

[ref79] Stuchi E. S. (2005). Adensamento de plantio: estratégia para a produtividade e lucratividade na citricultura. Rev. Ciênc. Prát. 16, 5–6.

[ref80] Stuchi E. S. Donadio L. C. (2000). Laranjeira ‘Folha-Murcha’. Jaboticabal: Funep (Boletim citrícola, 12).

[ref81] Torres C. M. García I. E. (2012). Nuevos cultivares de naranjo valencia para la isla de la juventud. Agricultura Orgánica 18, 28–30.

[ref82] U.S. Department of Agriculture (2021). FAS – Foreign Agricultural Service. Available at: https://www.fas.usda.gov (Accessed March 11, 2022).

[ref83] Vargas R. G. Gonçalves-Zuliani A. M. O. Croce Filho J. Carvalho S. A. Nocchi P. T. R. Nunes W. M. D. C. (2013). Avaliação da resistência de variedades de *Citrus* spp. à *Xanthomonas citri* subsp. *citri* na região Noroeste Paranaense, em condições de campo. Summa Phytopathol. 39, 235–241. doi: 10.1590/S0100-54052013000400001

[ref84] Wheaton T. A. Castle W. S. Whitney J. D. Tucker D. P. H. (1991). Performance of citrus scion cultivars and rootstock in a high-density planting. HortScience 26, 837–840. doi: 10.21273/HORTSCI.26.7.837

