# Supplementary material for: Late-Season Sweet Orange Selections Under Huanglongbing and Citrus Canker Endemic Conditions in the Brazilian Humid Subtropical Region
Source: Front Plant Sci. 2022 May 31;13:915889. doi: 10.3389/fpls.2022.915889 (PMC9205213; doi:10.3389/fpls.2022.915889)
Supplement: Supplementary file 1 [file Table_1.DOCX]

**Supplementary Table 1.** List of sequences and targeted genomic loci of primers used to identify ‘*Candidatus* Liberibacter asiaticus’ (*C*Las) in sweet orange (*Citrus ×sinensis*) plants.

| Primer set | Primer sequence (5´–3´) | Genomic locus | Amplicon size (bp) |
| --- | --- | --- | --- |
| A2/J5 | TAT AAA GGT TGA CCT TTC GAG TTT/ ACA AAA GCA GAA ATA GCA CGA ACA A | rplA/J | 703 |
| Oi1/Oi2c | GCG CGT ATG CAA TAC GAG CGG CA/ GCC TCG CGA CTT CGC AAC CCA T | 16S rDNA | 1,160 |
